# The role of bacterial transport systems in the removal of host antimicrobial peptides in Gram-negative bacteria

**DOI:** 10.1093/femsre/fuac032

**Published:** 2022-06-24

**Authors:** Jessica M A Blair, Kornelius Zeth, Vassiliy N Bavro, Enea Sancho-Vaello

**Affiliations:** College of Medical and Dental Sciences, Institute of Microbiology and Infection, University of Birmingham, Edgbaston, Birmingham, B15 2TT, United Kingdom; Department of Science and Environment, Roskilde University, Universitetsvej 1, 4000 Roskilde, Denmark; School of Life Sciences, University of Essex, Colchester, CO4 3SQ, United Kingdom; College of Medical and Dental Sciences, Institute of Microbiology and Infection, University of Birmingham, Edgbaston, Birmingham, B15 2TT, United Kingdom

**Keywords:** efflux pumps, antimicrobial peptides, antimicrobial resistance mechanisms, MtrCDE, AcrAB–TolC, Sap system

## Abstract

Antibiotic resistance is a global issue that threatens our progress in healthcare and life expectancy. In recent years, antimicrobial peptides (AMPs) have been considered as promising alternatives to the classic antibiotics. AMPs are potentially superior due to their lower rate of resistance development, since they primarily target the bacterial membrane (‘Achilles' heel’ of the bacteria). However, bacteria have developed mechanisms of AMP resistance, including the removal of AMPs to the extracellular space by efflux pumps such as the MtrCDE or AcrAB–TolC systems, and the internalization of AMPs to the cytoplasm by the Sap transporter, followed by proteolytic digestion. In this review, we focus on AMP transport as a resistance mechanism compiling all the experimental evidence for the involvement of efflux in AMP resistance in Gram-negative bacteria and combine this information with the analysis of the structures of the efflux systems involved. Finally, we expose some open questions with the aim of arousing the interest of the scientific community towards the AMPs—efflux pumps interactions. All the collected information broadens our understanding of AMP removal by efflux pumps and gives some clues to assist the rational design of AMP-derivatives as inhibitors of the efflux pumps.

## Teaser

This review describes how Gram-negative bacteria use efflux pumps as a mechanism of resistance against antimicrobial peptides (AMPs), including the efflux of AMPs to the extracellular space and the internalization of AMPs to the cytoplasm followed by proteolytic digestion. We have summarized the experimental evidence for the involvement of efflux in AMP resistance and combined this with analysis of the structure of the efflux systems involved and address the significant remaining open challenges and questions in the field.

## Introduction to antimicrobial peptides

Antibiotic resistance is a major global challenge that threatens the progress in healthcare and life expectancy. Although there are several antibiotics in preclinical and clinical trials, it is urgent to find new and more effective candidates to deal with this health emergency situation (Theuretzbacher et al. 2019, Butler and Paterson 2020).

Over the last decades, natural weapons such as bacteriocin proteins and peptides, endolysins, and antibodies have received substantial attention as potential clinical antimicrobials and as possible immune-modulating agents (Rios et al. 2016, Soltani et al. 2021). In particular, antimicrobial peptides (AMPs) are being considered as an alternative to the classical antibiotic approach. AMPs are a diverse group of peptides produced by multicellular organisms as a part of their first-line defence mechanism against pathogen invasion (Zasloff 2002, Wang and Wang 2004, Wang et al. 2022). AMPs can inhibit proinflammatory responses induced by lipopolysaccharides (LPS), act as adjuvants, modulate cytokine production, exert direct chemotactic action on neutrophils, macrophages, immature dendritic cells, mast cells, monocytes, and T-lymphocytes, or even activate endothelial cells to proliferate and form vessel-like structures in wound repair (Diamond et al. 2009).

**Figure 1.**
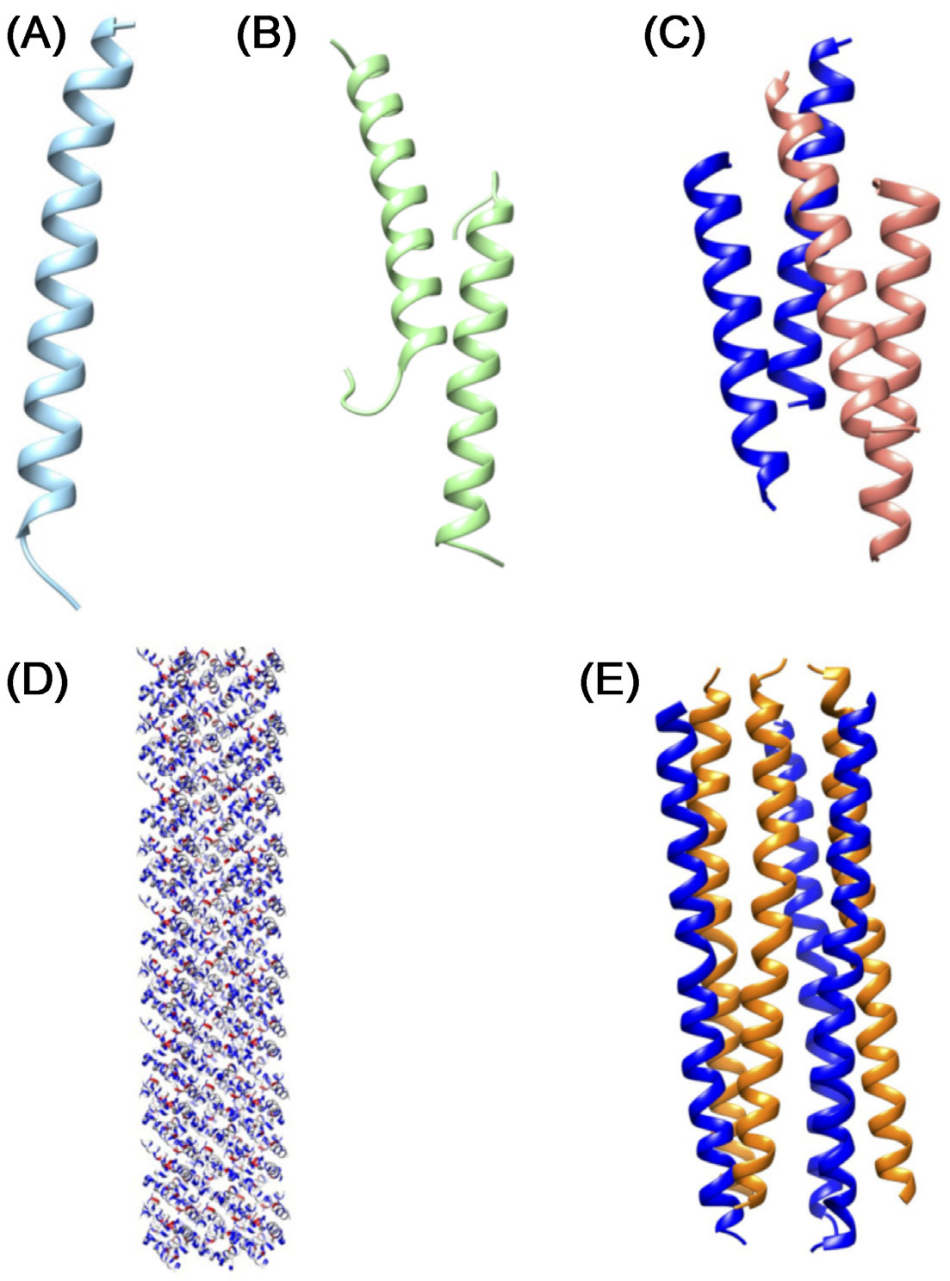
3D structures of full length and truncated LL-37 and dermcidin. (A)–(D) The different human LL-37 structures show their structural plasticity. (A) The monomeric structure of LL-37 (PDB 5NMN), (B) antiparallel dimer structure crystallized in the presence of detergents (PDB 5NNT), (C) tetrameric structure of LL-37 (7PDC), and (D) fiber of the core sequence of LL-37 peptide (residues 12–29; PDB 6S6M). (E) Hexameric channel structure of dermcidin (PDB 2YMK).

These small peptides (10–50 amino acids) are amphipathic molecules, mostly cationic (with a charge of +2 to +11), although anionic AMPs have also been reported (Schittek et al. 2001, Lai et al. 2007, Harris et al. 2009, Mahlapuu et al. 2016). Structurally, AMPs can be divided into linear α-helical, β-sheet, mixed, and linear extended/unfolded molecules (Table 1; Koehbach and Craik 2019). The linear α-helical group members typically contain one α-helix (e.g. LL-37, magainin, cecropin, and melittin). The β-sheet members contain at least two β-strands in their structure stabilized by two to four disulfide bridges (e.g. HD-5 and protegrin-1). The mixed AMPs contain both α- and β- structural elements (e.g. HBD-1). The linear extended structures do not exhibit any clear structural arrangement (e.g. indolicidin). In addition, AMPs can exhibit a huge variability in conformation and oligomerization, as seen by the different 3D structures/oligomers that the same AMP (e.g. LL-37) can adopt (Zeth and Sancho-Vaello 2021). Some of the AMPs show oligomeric structures such as LL-37 with dimers and tetramers or dermcidin as a hexameric channel (Fig. 1; Song et al. 2013, Sancho-Vaello et al. 2017, 2020).

**Table 1.** Structural properties and classification of the AMPs mentioned in this review.

AMP	Organism	Known biophysical properties and mode of action	PDB	Amino acids	Charge	References
**Ribosomal AMPs**						
Linear α-helical						
LL-37	The 18-KD protein hCAP-18 is the only cathelicidin found in humans so far. It consists of a cathelin-like domain and a C-terminal peptide called LL-37, which is released after a proteolytic event.	Disordered in water but helical structure in presence of ions, salts, detergents, or bacterial membranes. It is a very flexible peptide exhibiting multiple conformations and quaternary structures.	See different LL-37 3D structures and the corresponding PDBs in Fig. 1(A)–(D).	37	+6	Sørensen et al. (2001), Porcelli et al. (2008), Wang (2008), Morizane et al. (2010), Vandamme et al. (2012), Shahmiri et al. (2016), Sancho-Vaello et al. (2017, 2020), Engelberg and Landau (2020).
CRAMP	Cathelin-Related Antimicrobial Peptide is the mouse analogue of human LL-37 peptide. It is released from the cathelicidin proform after a proteolytic event.	α-helical structure as other cathelicidins. CRAMP-38 seems to be equivalent to CRAMP-2. In the AMP database, CRAMP is composed of 34 amino acids.	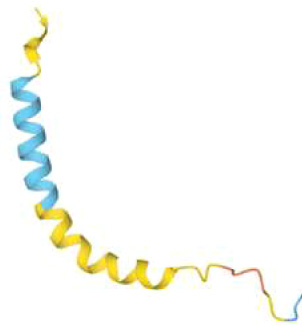 Alphafold prediction (entry P51437).	33(CRAMP-1) 38(CRAMP-2)	+6	Gallo et al. (1999), Kovach et al. (2012).
Cecropin	Cecropin was the first insect AMP isolated from the bacteria-challenged *Hyalophora cecropia* pupa.	Unstructured in aqueous solution but forming a high percentage of α-helical structure in the presence of LPS vesicles, or liposomes.	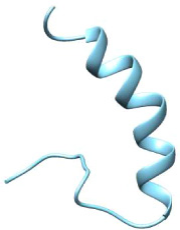 PDB: 2N92	35(Cecr.B) 31(Cecr.P1)	+7	Steiner et al. (1981), Hultmark et al. (1982), Wang et al. (1998), Bland et al. (2001).
Melittin	Melittin constitutes 50% of the dry weight of the honey bee venom.	Random coil configuration at low ionic concentration and neutral pH. It is a very flexible peptide exhibiting multiple conformations and quaternary structures (from monomer to tetramer).	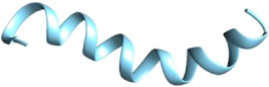 PDB: 6DST	26	+5	Altenbach and Hubbell (1988), Goto and Hagihara (1992), Son et al. (2007).
Snakin-1	Snakin-1 is a peptide isolated from potato tubers.	Mostly α-helical with six disulfide bonds between its 12 cysteines.	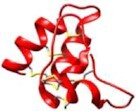 PDB: 5E5Q	63	+8	Segura et al. (1999)
**β-sheet**						
α-defensins	Human neutrophil peptides HNP-1,-2,-3,-4, and human enteric HD-5, -6. Defensins are synthesized as inactive precursors being activated through posterior proteolytic removal of the inhibitory propeptide.	Able to dimerize and form barrel-stave pores that span anionic membranes. A concentration-dependent equilibrium between monomers and higher oligomers has also been proposed.The structure of the membrane-bound HNP-1 showed a similar conformation to the water-soluble state, except for the turn connecting the β2 and β3 strands, supporting in this way a ‘dimer pore’ topology. They have three disulfide bonds.	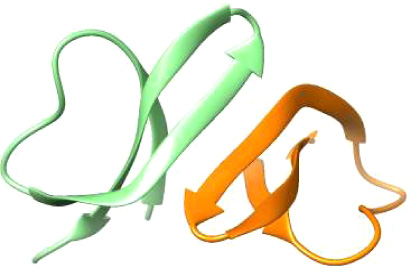 PDB: 1ZMM	30(HNP-1) 29(HNP-2) 30(HNP-3) 33(HNP-4) 32(HD-5,-6)	+3(HNPs) +4(HD-5) +2(HD-6)	Hill et al. (1991), Liu and Ganz (1995), Valore et al. (1996), Zhang et al. (2010), Shafee et al.(2017).
Protegrin-1	Protegrin-1 (PG-1) is synthesized in porcine leukocytes. PC-8 is a linearized synthetic variant lacking both disulfide bonds.	Ion channel-like structures with predominantly three to five subunits. The NMR structure suggested a membrane-inserted β-barrel enclosing a water pore. In the POPC/cholesterol membrane, the N and C strands of PG-1 cluster into tetramers. It has two intramolecular disulfide bonds.	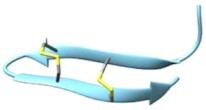 PDB: 1PG1	18	+7	Qu et al. (1997), Mani et al. (2006), Capone et al.(2010)
Tachyplesin-1	TP-1 is a 17 amino acid AMP extracted from the hemocytes of the horseshoe crab.	Structure stabilized by two cross-strand disulfide linkages, forming a β-hairpin structure, both in aqueous solution and in lipid-mimicking environments. It has two intramolecular disulfide bonds.	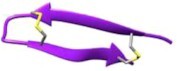 PDB: 2MDB	17	+7	Kawano et al. (1990), Kushibiki et al. (2014).
**Mixed**						
β-defensins	Human HBD-1,-2,-3,-4. Defensins are synthesized as inactive precursors being activated through posterior proteolytic removal of the inhibitory propeptide.There is an analog in chinchilla called cDB-1.	β-defensins are mostly β-sheets with a short Nt α-helix. Able to dimerize to form pores through anionic membranes. HBD-2 was found in solution mainly as dimers although it is likely to form higher-order oligomers either in higher concentrations that are induced by pathogen attack or in interactions with the lipid membranes of the bacterial cells. They have three disulfide bonds.	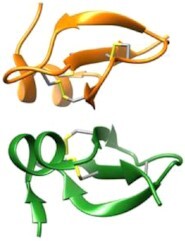 PDB: 1FD4	36(HBD-1) 41(HBD-2) 45(HBD-3) 50(HBD-4)	+4(HBD1) +7(HBD2) +11(HBD3) +6(HBD4)	Hoover et al. (2000), Harris et al. (2004), Shafee et al. (2017).
Thionins	The thionins are AMPs in plants.	Composed of two antiparallel α-helices and an antiparallel double-stranded β-sheet with three or four conserved disulfide linkages. They have three or four disulfide bonds.	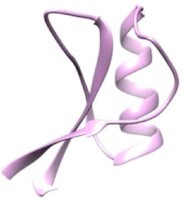 PDB: 1GPT	47	+9	Lucca et al. (2005), Nawrot et al. (2014).
Protamine	Protamines are short proteins that can contain up to 70% arginines found in the nuclei of sperm of different animal species. It participates in the packing of the DNA.	Protamine changes from a random coil to α-helix on binding tRNA.The DNA-bound protamine showed β-turns and α-helix, but no β-sheet was observed by infrared spectroscopy.They have two (bull) or none (piscine) disulfide bonds.	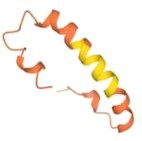 Alphafold prediction (entry P02319).	50–110	∼ +24	Wade et al. (1978), Aspedon and Groisman (1996), Roque et al. (2011).
**Linear extended**						
Indolicidin	This AMP was isolated from the neutrophil blood cells of cows.	Extended structure located in the membrane interface when forming complexes with vesicles and detergent micelles.	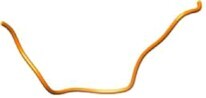 PDB: 1G89	13	+4	Rozek et al. (2000).
**Cyclic/complex topologies**						
Nisin	This lantibiotic is produced by *Lactococcus lactis* and is used as a food preservative. The two common forms of nisin are nisin A and Z, being recently isolated a new natural variant called nisin Q.	Structure consisting of two domains: an N-terminal domain containing three lanthionine rings, and a C-terminal domain containing two intertwined lanthionine rings. Nisin sequesters lipid II, resulting in prevention of cell wall biosynthesis, and forms nisin–lipid II complexes, which lead to pores in the bacterial cell membrane.	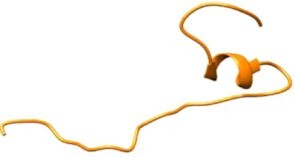 PDB: 1WCO	34	+3	Van Den Hooven et al. (1996), Wiedemann et al. (2001), Hsu et al. (2004).
**Nonribosomal AMPs**						
Polymyxin B	These AMPs are used as last-resort antibiotics for the treatment of MDR Gram-negative infections.	The structure of polymyxin B consists of a polycationic cyclic heptapeptide with a branched fatty acid tail. It is a mixture of four closely related components, only differing in the fatty acid moiety. Colistin and Polymyxin B share a common scaffold and present only minor changes in their constituent amino acids. Both of them interact with the LPS of the OM of Gram-negative bacteria displacing divalent cations and promoting membrane disruption. They also disrupt the inner bacterial membranes.	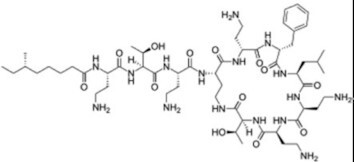	10	+6 (PXB, COL)	Hancock (1997), Orwa et al. (2001), Kwa et al. (2007), Zavascki et al. (2007), Deris et al. (2014), Ayoub Moubareck (2020).
Colistin (Polymyxin E)			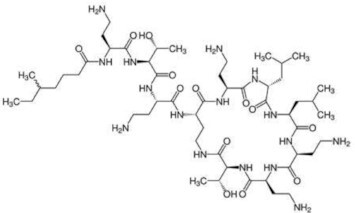			

In the interest of brevity, this review is focussed predominantly on the eukaryotic AMPs, however, the medical importance of prokaryotic bactericidal peptides is well-established. The ribosomally synthesized bacteriocin peptides often have high antimicrobial activity and include the subgroup of lanthionine containing peptide antibiotics known as lantibiotics (e.g. nisin; Table 1; Cotter et al. 2013). The lantibiotics are post-translationally modified and contain dehydrated amino acids (dehydrobutyrine and/or dehydroalanine) amongst other unusual amino acids (Willey and van der Donk 2007, Bierbaum and Sahl 2009). This has been recently reviewed in detail (Clemens et al. 2017, Kumariya et al. 2019). Another group of AMPs are the cyclic nonribosomal AMPs (e.g. colistin and polymyxin B). These AMPs are synthesized by bacteria (*Bacillus/Paenibacillus polymyxa*) and are currently used as a last-resort antibiotics for the treatment of MDR Gram-negative infections, but microbial resistance towards these antibiotics has already been reported (El-Sayed Ahmed et al. 2020). Even though these AMPs are not synthesized by multicellular organisms, the overview of the efflux of colistin and polymyxin B is included here with the aim of understanding whether efflux is a relevant mechanism of resistance to these AMP-based therapeutic agents.

Certain AMP properties such as size, amphipathicity, and especially their cationic nature allow them to target different molecules of the bacterial cell envelope and the cytoplasm, including the negatively charged LPS, the lipoteichoic acid (LTA) of the Gram-positive bacterial cell wall, phospholipids of the bacterial membranes, proteins, nucleic acids, or ribosomes (Ding et al. 2003, Malanovic and Lohner 2016, Macleod et al. 2019, Martynowycz et al. 2019).

The main killing mechanism used by the AMPs is the disruption of bacterial membranes through initial electrostatic interactions with LPS or LTA in the outer membrane (OM) or cell wall, respectively, after a threshold concentration of accumulated AMP is reached (Ding et al. 2003, Malanovic and Lohner 2016). Classically, the cytoplasmic membrane disruption mechanisms have been divided into the detergent-like carpet model, the toroidal pore model, and the barrel-stave model (Zasloff 2002, Brogden 2005, Bechinger and Lohner 2006). In the carpet model (e.g. cecropin B), the disruption of the membrane occurs after the formation of a layer of peptide monomers on the membrane surface destabilizing its phospholipid packing and leading to its disintegration (Gazit et al. 1996). In the toroidal pore model (e.g. melittin), the peptides interact with the head of the phospholipids in order to form a combined peptide–lipid pore (Lee et al. 2013). In the barrel-stave model (e.g. dermcidin), the peptides form a pore exclusively composed of peptides (Mihajlovic and Lazaridis 2010, Song et al. 2013, Sancho-Vaello et al. 2020). However, there are now also known to be intermediate mechanisms and combinations of these mechanisms (Wimley 2010, Nguyen et al. 2011, Sancho-Vaello et al. 2017).

The formation of pores in membranes can be transient or stable, as shown by the concentration-dependent pore formation in melittin. At nanomolar concentrations, it induces transient pores that allow transmembrane conduction of atomic ions, but not leakage of glucose or larger molecules. Beyond a critical peptide/lipid ratio, pores become stable and lead to leakage of cellular contents, the loss of transmembrane potential, and death of the bacteria (Terwilliger and Eisenberg 1982, Matsuzaki et al. 1997, Lee et al. 2013). If the OM pores are transient or their number is low, the peptides can access the periplasm, where they can interact with other proteins and/or accumulate on the surface of the inner membrane (IM). After a peptide/phospholipid threshold is reached in the cytoplasmic membrane, the peptides can oligomerize, form pores and access the cytoplasm where they can interact with cytoplasmic targets including ribosomes and nucleic acids (Graf et al. 2017, Cardoso et al. 2019). Proline-rich AMPs (e.g. oncocin, Api137) have been shown to bind to ribosomes and inhibit protein synthesis *in vivo* and *in vitro* (Krizsan et al. 2015, Mardirossian et al. 2018). Specifically, the Oncocin and Onc112 allow translation initiation but prevent the transition into the elongation phase (Seefeldt et al. 2015). Others, such as the Api137 arrests terminating ribosomes (Florin et al. 2017). Some AMPs (e.g. indolicidin) induce filamentation in *Escherichia coli* cells as a result of DNA synthesis inhibition (Subbalakshmi and Sitaram 1998). In this way, AMPs can interfere with vital intracellular processes, such as cell wall or protein synthesis (Le et al. 2017).

Although several mechanisms of AMP resistance have been shown *in vitro* (see the section 'Mechanisms of AMP resistance in Gram-negative bacteria' in this review) and by using *in vivo* models of infection (Mount et al. 2010, Hobbs et al. 2011, 2013, Bauer and Shafer 2015), it has been shown that AMP resistance evolves at a much lower rate than to antibiotics, except for the nonhost defence peptide colistin (Peschel and Sahl 2006, Spohn et al. 2019). Recently, other constraints on the evolution of AMP resistance have been proposed to explain their low rate of appearance. These evolutionary constraints are related to fitness trade-offs, functional compatibility, and the small fraction of AMP resistance genes linked to mobile genetic elements (Jangir et al. 2021). Specifically, it was shown that whereas AMP resistance genes are widespread in the gut microbiome, their rate of horizontal transfer is lower than that of antibiotic resistance genes. By gut microbiota culturing and functional metagenomics it was revealed that AMP resistance genes originating from phylogenetically distant bacteria have only a limited potential to confer resistance in *E. coli* (Kintses et al. 2019). Related to the fitness trade-offs, it was shown that increased expression of *mcr-1* (a lipid A modifying enzyme that confers resistance to colistin) results in decreased growth rate, cell viability, competitive ability, and significant degradation in cell membrane and cytoplasmic structure (Yang et al. 2017).

A second advantage of using AMPs as antimicrobial agents is their ability to target the challenging nonpermeable double membrane of Gram-negative bacteria (Fjell et al. 2011). In particular, the synergy of some AMPs when used in combination with the classical antibiotics have opened the possibility for decreasing their therapeutic dose (Steenbergen et al. 2009, Wu et al. 2020, Kampshoff et al. 2019, Pizzolato-Cezar et al. 2019, Ruden et al. 2019). For example, the AMP DP7 in combination with vancomycin or azithromycin was more effective, especially against highly antibiotic-resistant strains (Wu et al. 2017).

All AMPs used currently in authorized treatments belong to the nonribosomally synthesized group (e.g. polymyxin B and colistin). However, none of the more than 3000 identified ribosomally encoded AMPs have been approved by the FDA (Browne et al. 2020, Liu et al. 2019, Chen and Lu 2020) because of issues with the AMP sensitivity to environmental conditions in particular proteolysis (Mahlapuu et al. 2016), the high production costs of the chemical modifications needed to overcome their instability (e.g. use of D-amino acids, macrocyclization by using disulfide bonds, and incorporation of noncanonical amino acids), and the toxicity against mammalian cells *in vivo* (Haney and Hancock 2013, Koo and Seo 2019). Despite these issues, the number of AMPs with activities related to membrane disruption, immunomodulation, or inhibition of intracellular functions is increasing in clinical and preclinical development (Browne et al. 2020, Koo and Seo 2019).

## Mechanisms of AMP resistance in Gram-negative bacteria

In order to avoid AMP accumulation on their surface and consequent membrane pore formation, bacteria have to protect their exposed surfaces (cell wall, OM, and IM) and the potential cytoplasmic targets (DNA and ribosomes) against AMP attachment. To do this, bacteria employ different defence mechanisms including extra- or intracellular proteolytic attack, the alteration of membrane charge and fluidity of the bacterial membrane, the use of extracellular matrices to entrap AMPs, and the active removal of AMPs through efflux pumps. Detailed reviews of other AMP resistance mechanisms have been recently published (Koprivnjak and Peschel 2011, Matamouros and Miller 2015, Cole and Nizet 2016, Joo et al. 2016, Bechinger and Gorr 2017) so in this review, we will only briefly describe the mechanisms employed by Gram-negative bacteria to resist the action of AMPs, but provide an in depth view on the available data concerning AMP efflux.

(a) Proteolysis by extracellular and intracellular proteases

**Figure 2.**
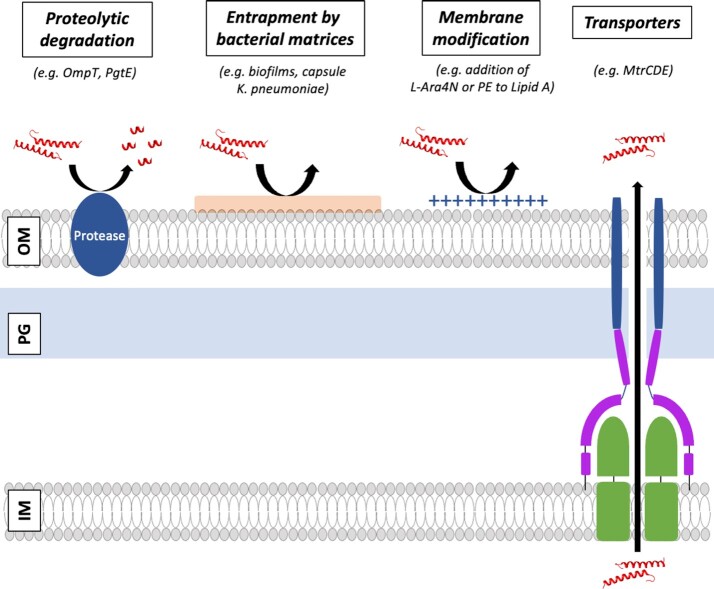
Mechanisms of AMP resistance in Gram-negative bacteria, including (A) proteolysis by extracellular and intracellular proteases; (B) entrapment by bacterial biofilms and other extracellular matrices; (C) modification of charge and fluidity of bacterial membranes; and (D) removal of AMPs through transporters. The schematic representation and localization of the PG is based on (Ma et al. 2021).

In Gram-negative bacteria, several proteases have been shown to confer AMP resistance by cleaving AMPs at the OM (Fig. 2). For example, *E. coli* OmpT, *Salmonella enterica* serovar Typhimurium PgtE, and *Yersinia pestis* Pla, belong to the omptin family of aspartate proteases and can cleave LL-37, C18G, CRAMP, and protamine (Galván et al. 2008, Guina et al. 2000, Stumpe et al. 1998, Thomassin et al. 2012). In *Proteus mirabilis*, the metalloprotease ZapA cleaves the human HBD-1, LL-37, and PG-1 (Belas et al. 2004). In *Burkholderia cenocepacia*, two zinc-dependent metalloproteases (ZmpA and ZmpB) can cleave and inactivate LL-37 and HBD-1, respectively (Kooi and Sokol 2009). The *Pseudomonas aeruginosa* elastase completely degrades and inactivates LL-37 (Schmidtchen et al. 2002). Also, some proteases secreted by *Porphyromonas gingivalis* and *Prevotella spp*. can cleave cecropin B and brevinin (Devine et al. 1999). In *S*. Typhimurium and *Haemophilus influenzae*, the cytoplasmic proteases can also degrade AMPs via Sap transport, as explained in the section 'Sap system: importing and degrading AMPs as mechanism of resistance' of this review.

(b) Entrapment of AMPs by bacterial biofilms and other extracellular matrices

Bacterial biofilms and other extracellular matrices can hinder the AMP attachment to the bacterial surface, by decreasing the penetration of AMPs through the matrix (Fig. 2; Mah and O'Toole 2001). In *P. aeruginosa*, the production of alginate polysaccharide induces aggregation in some AMPs (Chan et al. 2004, Foschiatti et al. 2009). The polysaccharide intercellular adhesin (PIA) produced by *E. coli* was shown to be responsible for HBD-3, LL-37, and dermcidin resistance (Wang et al. 2004).

Also, the capsule of *Klebsiella pneumoniae* is responsible for conferring resistance against polymyxin B, HNP-1, lactoferrin, and protamine likely by hindrance and electrostatic trapping (Campos et al. 2004). The same mechanism seems to work in the *Neisseria meningitidis* capsule that prevents AMP surface binding of LL-37, protegrins, defensins, polymyxin B, LL-37, and CRAMP (Jones et al. 2009, Spinosa et al. 2007).

Another approach to entrap the cationic AMPs is to perform a proteolytic release of negatively charged elements belonging to the host epithelial cells. Some proteases in *P. aeruginosa* can degrade the proteoglycan decorin releasing dermatan sulfate, which can bind and inactivate the α-defensin HNP-1 (Schmidtchen et al. 2001).

(c) Modification of charge and fluidity of bacterial membranes

Perhaps the most common strategy to decrease the ionic attraction between the cationic AMPs and the negatively charged elements of the bacterial membranes is the bacterial surface charge modification (Fig. 2). The Gram-negative double membrane is a challenging structure to penetrate. It consists of an asymmetric OM with an inner leaflet containing phospholipids, and an outer leaflet mostly composed of LPS. Between the OM and the IM, there is a periplasmic space containing a thin layer of peptidoglycan. The IM is a phospholipid bilayer composed of phosphatidylethanolamine (PEA), phosphatidylglycerol (PG), phosphatidylserine (PS), and cardiolipin (Silhavy 2010).

One mechanism that bacteria employ to decrease the negative net charge of LPS is to add the positively charged 4-amino-4-deoxy-L-arabinose (L-Ara4N) or phosphoethanolamine (PEA) moieties to lipid A. In *Salmonella spp*, this modification is regulated by the two-component system PmrAB, which senses the AMP presence *in vivo* and expresses *pmrC* and *pmrEHFIJKLM* (Gunn and Miller 1996, Gunn et al. 2000, Tamayo et al. 2005). Specifically, in *Salmonella*, the PmrA-dependent modification of lipid A was shown to be responsible for polymyxin B resistance (Lee et al. 2004). The enhancement of AMP resistance by the addition of L-Ara4N has been also shown in *P. mirabilis* (McCoy et al. 2001), *Yersinia pseudotuberculosis* (Marceau et al. 2003)*, K. pneumoniae* (Cheng et al. 2010), and *P. aeruginosa* (Moskowitz et al. 2004). The addition of pEtN to dephosphorylated lipid A also involves an increase in AMP resistance. This has been shown in *Helicobacter pylori* under the regulation of the *lpxEHP* genes and showed an increase in MIC of polymyxin B (Tran et al. 2006). The polymyxin B resistance was also observed in *Neisseria gonorrhoeae* and *N. meningitidis* by the mediation of the *lptA* gene (Lewis et al. 2009, Tzeng et al. 2005). This effect was also shown *in vivo*, by inoculating mice and men with mixtures of *N. gonorrhoeae* wild-type (WT) and an isogenic mutant lacking the PEA transferase LptA (Hobbs et al. 2013). In *Campylobacter jejuni*, mutants in *waaF, cstII, galT*, or *lgtF* genes abolished the lipooligosaccharide/LPS production, becoming resistant to polymyxin B or HD-5 (Keo et al. 2011, Naito et al. 2010).

Also, the addition of palmitoyl groups to Lipid A by the acyltransferase PagP increases the resistance to AMPs by reducing the OM permeability. In *S. Typhimurium*, this acylation is regulated by the two-component system PhoPQ, decreasing the fluidity and permeability of the OM (Bishop et al. 2000, Dalebroux and Miller 2014, Guo et al. 1998). This strategy promoted the resistance against AMPs such as C18G, protegrin, polymyxin B, LL-37, and magainin II (Guo et al. 1998). In *H. influenzae*, the lipooligosaccharide acylation mediated by the *htrB* gene was shown to be responsible for the resistance against HBD-2 (Starner et al. 2002). The addition of phosphorylcholine to the oligosaccharide portion of LPS confers resistance against LL-37 in *H. influenzae*(Lysenko et al. 2000). Also, in *K. pneumoniae* the gene *lpxM* increases susceptibility to AMPs since it enhances OM permeability (Clements et al. 2007).

In *P. aeruginosa*, alanylation of PG (which does not change the overall net charge) by the multiple peptide resistance factor MprF has been reported as another mechanism to disrupt the ability of protamine to bind and disrupt the bacterial membrane (Klein et al. 2009). In *Salmonella, a pmrAB*-regulated mechanism involving the change of O-antigen length has been linked to AMP resistance. In this way, mutants with long O-antigen chains showed a mild increase in susceptibility to polymyxin B (Farizano et al. 2012).

(d) Removal of AMPs mediated by transporters

**Figure 3.**
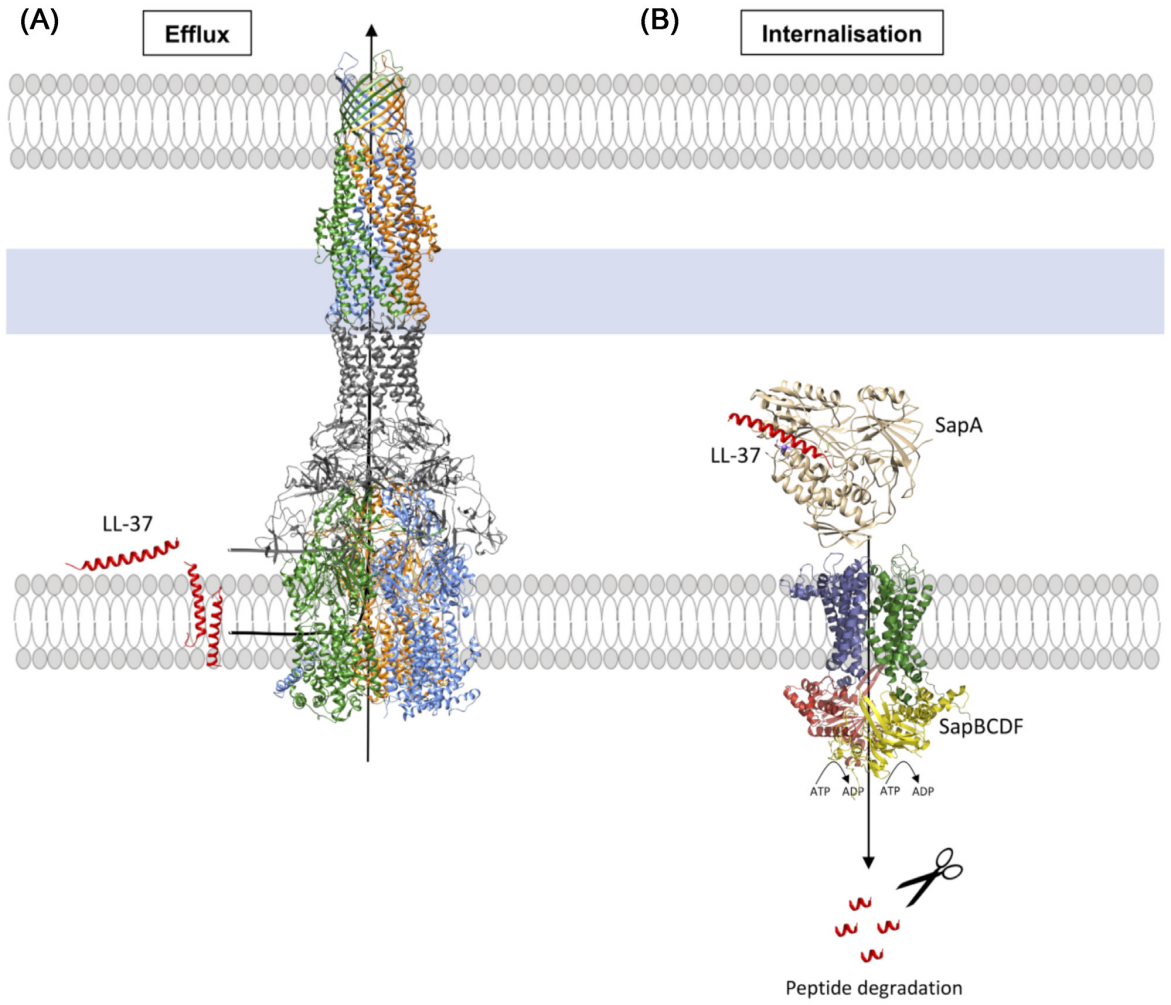
Transport-related mechanisms of AMP resistance. Efflux pumps such as MtrCDE and AcrAB–TolC can efflux AMPs out of bacterial cells, while the Sap transporter introduces the AMP into the cytoplasm in order to be degraded. (A) The structure of the *E. coli* AcrAB–TolC tripartite pump (PDB 5NG5). The protomers of AcrB and TolC are shown in green, orange, and blue, respectively. AcrA is coloured in dark grey. (B) Sap transporter. The figure contains a model of the *E. coli* Sap transporter (this review) showing SapB in blue, SapC in green, SapD in red, and SapF in yellow. This model has been based on the AlphaFold outputs for individual proteins and the assembly of the tetramer has been guided by the structure of the bacterial alginate ABC transporter (importer) AlgM1M2SS (a heterotetramer of AlgM1, AlgM2, and AlgS; PDB 4XTC), as per (Kaneko et al. 2017). This is a type I ABC transporter according to the latest nomenclature from (Thomas et al. 2020). The figure also contains the recently solved 3D structure of *H. influenzae* SapA (7OFZ). The AMP LL-37 (PDB 5MNM) is shown in ribbon representation. The figure was prepared in UCSF Chimera (Pettersen et al. 2004).

If the previously explained mechanisms fail and AMPs accumulate on the surface of the bacterial membranes, they can change their conformation after contact with phospholipids and, after reaching a threshold, access the periplasm or cytoplasm via transient pore formation (Melo et al. 2009). In this case, the bacteria still have a last resort mechanism to remove them. This mechanism is conducted by efflux pumps and transporters normally responsible for the introduction of nutrients and the efflux of harmful molecules from the bacterial cytoplasm (Figs. 2 and 3; Alav et al. 2021, Henderson et al. 2021). Efflux pumps are an important mechanism of AMR because they export antimicrobials to keep the bacterial intracellular concentrations below toxic levels (Blair et al. 2014, Colclough et al. 2020). In terms of transport, bacteria have two main strategies for dealing with AMPs: (1) pump them out of the bacterial cell or (2) transport them into the cytoplasm to be degraded by cytoplasmic proteases (as done by the Sap transporter; Fig. 3). Both strategies will be discussed here in detail.

## Efflux as a mechanism of AMP resistance in Gram-negative bacteria

Efflux pumps are molecular machines able to export a wide range of antimicrobials and metabolites (Henderson et al. 2021), and multiple lines of evidence implicate them also in clearance of AMPs (Eswarappa et al. 2008, Feng et al. 2003, Lister et al. 2012, Shafer et al. 1998, Clemens et al. 2017, Honeycutt et al. 2020, Wang et al. 2016). Their capacity to recognize a broad range of structurally different compounds may be explained by their structural flexibility and multiple binding sites (Alav et al. 2021).

There are seven generally recognized classes of efflux pumps:

1. The ATP-binding cassette (ABC) superfamily.
2. The resistance–nodulation–division (RND) superfamily.
3. The major facilitator (MFS) superfamily.
4. The multidrug and toxic-compound extrusion (MATE) family.
5. The drug/metabolite transporter superfamily (DMT) including the small multidrug resistance (SMR) family.
6. The proteobacterial antimicrobial compound efflux (PACE) family.
7. the p-aminobenzoyl-glutamate transporter (AbgT) family.

The two main efflux pump families involved in AMP resistance in Gram-negative bacteria are the ABC-transporter and the RND efflux pump superfamilies (Table 2). Some members of the MFS family have also been proposed to be able to confer the AMP resistance phenotype.

**Table 2.** Transporter systems reported to confer AMP resistance in Gram-negative bacteria to date. PG-1, protegrin-1; PXB, polymyxin B; CO, colistin; cBD-1, chinchilla β-defensin-1; CRAMP-38, mouse CRAMP; PRO, protamine; POLY, polyphemusin II; TAC, tachytegrin. The α-defensins include HNP-1, -2, -3, -4, HD-5, the β-defensins HBD-1, -2,-3,-4, and the rabbit defensins NP-1, -2.

Transporter family	Transportersystem	*Microorganism*	Substrates	No substrates	References
RND	MtrCDE	*N. gonorrhoeae*	LL-37, PG-1, PC-8	HNP-2, NP-2	Shafer et al. (1998)
			LL-37, CRAMP-38		Warner et al. (2008)
			LL-37		Handing et al. (2018)
			CO, PXB		Chitsaz et al. (2019)
		*N. meningitidis*	LL-37, PG-1, PXB		Tzeng et al. (2005)
		*Haemophilus ducreyi*	LL-37, HBD-2,-3,-4	HNP-1, -2, HD-5	Rinker et al. (2011)
	AcrAB–TolC	*K. pneumoniae*	PXB, HNP-1, HBD-1, HBD-2		Padilla et al. (2010)
		*Y. pestis*	PXB		Lister et al. (2012)
		*E. coli*		LL-37, PXB, HNP-1, -2, -3, HD-5, HBD-2	Rieg et al. (2009)
			LL-37, HBD-1, HNP-2*, PXB		Warner and Levy (2010)
			PRO		Weatherspoon-Griffin et al. (2014)
	MexAB–OprM	*P. aeruginosa*		LL-37, PXB, HNP-1, -2, -3, HD-5, HBD-2, -3	Rieg et al. (2009)
				PXB	Masuda et al. (2000)
	MexCD–OprJ	*P. aeruginosa*		PXB	Masuda et al. (2000)
	MexXY-OprM	*P. aeruginosa*		PXB	Masuda et al. (2000)
	CmeDEF	*C. jejuni*	PXB	PRO	Akiba et al. (2006)
	VexAB–TolC	*Vibrio cholerae*	PXB		Bina et al. (2008)
	H239_3064	*K. pneumoniae*	CO		Cheng et al. (2018)
ABC	Sap	*Haemophilus ducreyi*	LL-37	HNP-1, HNP-2, HD-5 & HBD-2, -3 and -4	Mount et al. (2010)
			LL-37	HD-5, HNP-2, HBD-2	Rinker et al. (2012)
		*S*. Typhimurium	PRO, melittin		Parra-Lopez et al. (1993)
			PRO, melittin	cecropin *P*-1, magainin-2, NP-1	Groisman et al. (1992)
		NTHI	cBD-1		Mason et al. (2005)
			cBD-1, HBD-3, LL-37		Mason et al. (2006)
			HBD-1, -2, -3, LL-37, HNP- 1, melittin		Mason et al. (2011)
			LL-37, HBD-3		Shelton et al. (2011)
		*Erwinia chrysanthemi*	snakin-1, α-thionin	Plant defensins, PRO	López-Solanilla et al. (1998)
		*E. coli*		LL-37	Sugiyama et al. (2016)
		*P. mirabilis*	PXB	PG-1, nisin, IB-367, POLY, TAC	McCoy et al. (2001)
		*Actinobacillus pleuropneumoniae*	PR-39		Xie et al. (2017)
		*Vibrio fischeri*		LL-37, PXB, PRO, CP11CN, CP26, CP28, CP29	Lupp et al. (2002)
	yejABEF	*Brucella melitensis*	PXB		Wang et al. (2016)
		*Salmonella*	PXB, melittin, PRO, HBD-1, -2		Eswarappa et al. (2008)
	MacAB–TolC	*Salmonella*	C18G		Honeycutt et al. (2020)
MFS	RosAB	*Yersinia enterocolitica*	PXB, cecropin P1, melittin		Bengoechea and Skurnik (2000)
	EmrAB–TolC	*E. coli*	PRO		Weatherspoon-Griffin et al. (2014)
	EmrAB	*Acinetobacter baumannii*	CO		Lin et al. (2017)
K + transporters (SKT)	TrkG/H–TrkA	*Vibrio vulnificus*	PRO, PXB		Chen et al. (2004)

* In Warner and Levy (2010), only the *tolC* mutant was slightly susceptible to HNP-2, while the *acrAB* mutant was not susceptible to this AMP.

The AMPs included in Table 2 exhibit one of the three classical mechanisms of membrane disruption (barrel-stave, toroidal, and carpet) or a combination of them. No correlation between mechanism of membrane disruption and ability to be efflux by the efflux pumps/transporters have been found by the authors of this review.

### The ABC superfamily

ABC transporters are ubiquitous to all domains of life, and are considered the most abundant transporters on Earth (Alav et al. 2021, Elbourne et al. 2017). The ABC transporters can transport a broad range of substrates, including metabolites, vitamins, amino acids, lipids, peptides, ions, and drugs (Rees et al. 2009, Thomas and Tampé 2018). Based on their sequence homology and architecture, ABC transporters can be divided into seven types performing different physiological functions (Thomas and Tampé 2020, Thomas et al. 2020).

ABC transporters use ATP hydrolysis as their primary energy source (Lubelski et al. 2007), sharing a common modular architecture including two highly conserved nucleotide-binding domains (NBDs) that contain the conserved Walker A and Walker B motifs, and two variable transmembrane domains (TMDs) forming the translocation pathway (Rees et al. 2009, Schneider and Hunke 1998). In bacteria and archaea, the four domains are often distinct subunits, or they are fused into homo- or heterodimerizing half-transporters composed of one NBD and one TMD (Thomas and Tampé 2020). These seven types are present in bacteria acting as importers (types I–III), exporters (types IV and V), extractors (type VI), or operating as tripartite efflux pumps (VII category). Recently the cryo-EM structure of the MlaFEDB complex, involved in phospholipid transport across the bacterial envelope, has revealed distant relationships to other ABC transporters, suggesting that a separate group should be added to the ABC transporters classification (Coudray et al. 2020, Malinverni and Silhavy 2009).

Regarding the ABC importers, the type I and II conserve the overall topology of ABC transporters having 5–6 or 10–12 helices in their TMDs, respectively. TMDs and NBDs dimerize and assemble the minimal unit of the importer. Both type I and type II transporters utilize a substrate binding protein (SBP), located in the periplasm of the Gram-negative bacteria, to recognize and deliver substrates to its cognate ABC transporter located in the IM (Tanaka et al. 2018). Similar to type I and II transporters, type III transporters [also called energy-coupling factor (ECF) transporters] consist of two NBDs (EcfA and EcfA´), a TMD (EcfT), and a substrate-binding component (EcfS). The EcfA–EcfA´–EcfT complex forms the energizing module while the EcfS component is an integral membrane protein that binds substrate with nanomolar affinity (Duurkens et al. 2007, Hebbeln et al. 2007). In the view of the rapid advances in structural biology of the ABC transporters, their classification has been frequently revised, e.g. by Rice et al. (2014), and more recently by Thomas et al. (2020), which we would like to direct the reader to for further details.

The ABC-transporters involved in AMP transport present different structural architectures including tripartite structures in which the IM ABC-transporter associates with an accessory periplasmic protein and an OM protein, as in the MacAB–TolC transporter (Kobayashi et al. 2001). The Sensitive-to-antimicrobial-peptides SapABCDF transporter has been shown to act as the main transporter of AMPs into the cytoplasm in nontypeable *H. influenzae* (NTHI), *Haemophilus ducreyi, S*. Typhimurium*, Erwinia chrysanthemi, P. mirabilis*, and *Actinobacillus pleuropneumoniae*, while the *E. coli* orthologue has been associated with putrescine transport (Fig. 3B and Table 2; Groisman et al. 1992, López-Solanilla et al. 1998, Mason et al. 2005, 2006, 2011, McCoy et al. 2001, Mount et al. 2010, Parra-Lopez et al. 1993, Rinker et al. 2012, Shelton et al. 2011, Sugiyama et al. 2016, Xie et al. 2017). A subsequent proteolytic degradation step by bacterial cytoplasmic proteases was shown (Handing et al. 2018, Mason et al.^2005^, ^2006^, ^2011^, Mount et al. 2010, Rinker et al. 2012, Shelton et al. 2011). In addition, the YejABEF transporter in *Salmonella* and *Brucella* and the tripartite pump MacAB–TolC in *Salmonella* appear to confer AMP resistance (Table 2; Eswarappa et al. 2008, Honeycutt et al. 2020, Wang et al. 2016).

### The RND Superfamily

This superfamily has members in all three domains of life and contains the most clinically relevant efflux pumps associated with the MDR phenotype in bacteria (Saier et al. 2006). RND pumps are Proton Motive Force (PMF) driven and depend on the pH gradient over the IM. The export of amphiphilic and hydrophobic substrates is governed by the hydrophobic–amphiphilic efflux (HAE–RND) family, while the efflux of heavy metals relies on the heavy metal efflux (HME–RND) family (Alav et al. 2021, Colclough et al. 2020, Klenotic et al. 2021). The HAE family members are IM proteins composed of ∼1000 amino acids and organized into 12 transmembrane helices (TMHs) with two large periplasmic loops between helices 1 and 2 and 7 and 8 (Colclough et al. 2020). In order to export AMPs out of the cell, these IM proteins form a tripartite assembly alongside members of the periplasmic adaptor protein (PAP) family and an OM protein channel belonging to the outer membrane factor (OMF) family, such as TolC (Alav et al. 2021, Colclough et al. 2020, Kobylka et al. 2020). Experimental evidence shows a stoichiometry of 3:6:3, comprising an IM trimer, an accessory protein hexamer and a TolC trimer (Fig. 3; Du et al. 2014, Janganan et al. 2011, Wang et al. 2017). In some Gram-negative bacteria, notably Enterobacteriaceae, the drug exporting RND pumps associate with accessory proteins such as AcrZ in the case of the AcrAB–TolC efflux pump (Hobbs et al. 2012). AcrZ and its homologues seem to induce conformational changes to AcrB that alter drug specificity (Du et al. 2020), and is speculated to play a modulatory role (Henderson et al. 2021). A relay network of transporters has been proposed in which IM proteins belonging to the MFS and SMR families act in the IM by transporting toxic compounds from the cytoplasm, while RND-based tripartite efflux pumps remove these compounds from the periplasmic space out of the cell (Tal and Schuldiner 2009).

Tripartite efflux pumps of the RND superfamily include AcrAB–TolC in *E. coli* (Fig. 3A);*Pseudomonas* Mex systems (MexAB–OprM, MexCD–OprJ, MexEF–OprN, and MexXY–OprM), and *Acinetobacter* Ade systems (AdeABC and AdeIJK) (Alav et al. 2021, Colclough et al. 2020, Klenotic et al. 2021). Among the members of the RND family, the *multiple transferable resistance* (*mtr*) operon (Hagman et al. 1995) coding for the tripartite pump MtrCDE has been shown to be important for AMP resistance in *N. gonorrhoeae, N. meningitidis*, and *H. ducreyi* as well as AcrAB–TolC in *Klebsiella* and *Y. pestis* (Table 2; Chitsaz et al. 2019, Handing et al. 2018, Lister et al. 2012, Padilla et al. 2010, Rinker et al. 2011, Shafer et al. 1998, Tzeng et al. 2005, Warner et al. 2008). The role of AcrAB–TolC in *E. coli* is also discussed in this review, since its involvement in AMP resistance has been debated (Rieg et al. 2009, Warner and Levy 2010, Weatherspoon-Griffin et al. 2014). Other RND pumps shown to confer AMP resistance include VexAB–TolC from *Vibrio cholerae*, CmeDEF in *C. jejuni*, and a new RND pump found in *K. pneumoniae* (named H239_3064), which has been related to colistin resistance (Table 2; Akiba et al. 2006, Bina et al. 2008, Cheng et al. 2018).

MtrCDE is composed of the PAP MtrC that bridges between the OM channel MtrE, and the IM-transporter MtrD (similar to AcrAB–TolC in Fig.   3A). The stoichiometry of the pump has been shown to be MtrD_3_–MtrC_6_–MtrE_3_ (Janganan et al. 2011). MtrCDE is directly regulated by the TetR family protein MtrR (Beggs et al. 2019). This efflux pump is particularly important in *N. gonorrhoeae* and *H. influenzae* as these microorganisms only contain a single RND efflux system, which is unusual among the Gram-negative bacteria (Maness and Sparling 1973, Zwama et al. 2019). This importance is further highlighted as the ectopic expression of *mtrR*, can restore the sensitivity of *N. gonorrhoeae* to previously used antibiotics (Chen et al. 2019).

Members of the OMF family of proteins, such as MtrE and TolC, form an elongated homotrimeric channel-tunnel, which is embedded in the OM using a beta-barrel similar to the porin-fold, while a large α-helical periplasmic domain extends nearly 130 Å long reaching the peptidoglycan layer (Koronakis et al. 2000, Lei et al. 2014). The periplasmic end of the OMF channels is sealed by specific charged interactions, which are thought to be broken upon engagement with the PAP partner (Bavro et al. 2008, Tamburrino et al. 2017). For MtrE, the stabilization of the channel in an open state seems to be related to the direct interaction with MtrC, similar to the stable assembled tripartite systems AcrAB–TolC and MexAB–OprM observed by cryo-EM microscopy (Wang et al. 2017; Tsutsumi et al. 2019; Glavier et al. 2020) and cryo-tomography *in situ* (Chen et al. 2021, Shi et al. 2019).

The PAP-component of the pumps—including MtrC, AcrA, and MexA, have a multidomain structure that can be likened to a ‘beads on a string arrangement’, including a long α-hairpin domain, a lipoyl domain, a β-barrel domain, and a membrane-proximal domain (Akama et al. 2004, Higgins et al. 2004, Mikolosko et al. 2006, Symmons et al. 2009, 2015).

**Figure 4.**
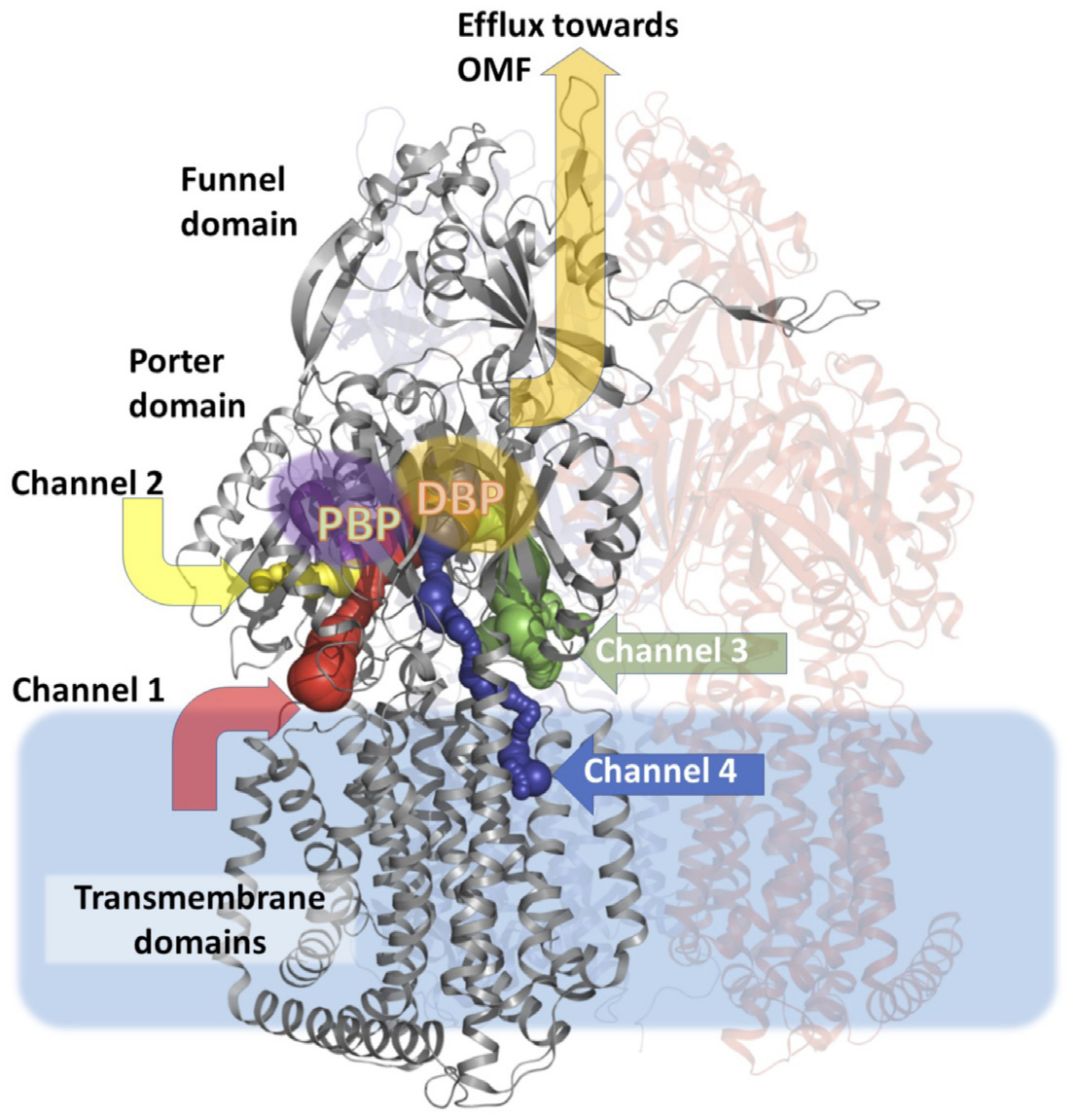
General organization of RND transporter trimer, shows oligomeric structure and principal substrate pathways. The distal binding pocket (DBP) and proximal binding pocket (PBP), as well as the entrance to channels 1–4 are indicated based on the *E. coli* AcrB structure [PDB ID: 5JMN; (Oswald et al. 2016)]. The L-protomer of the RND-transporter is shown and annotated, with the neighbouring protomers semitransparent. The blue rectangle delineates approximate limits of the IM.

The RND component of the pump, e.g. MtrD, (Bolla et al. 2014, Chitsaz et al. 2019, Fairweather et al. 2021, Lyu et al. 2020, Murakami et al. 2002, Nakashima et al. 2013, Sennhauser et al. 2009), is a trimeric transporter, each protomer of which contains 12 TMHs. MtrD shares 48.9% sequence identity with the homologous *E. coli* AcrB protein (Fig. 4). MtrD exhibits a large periplasmic domain, which is formed from two lobes that are spliced between TM1/TM2 and TM7/TM8, respectively. The resulting periplasmic domain can be divided into six subdomains: four of which form the porter domain (PN1, PN2, PC1, and PC2), while two (FN and FC) form the funnel (formerly OMF-docking) domain of each protomer (Bolla et al. 2014, Fairweather et al. 2021). The porter domain recognizes and transports the pump substrates, which bind within it to the so-called proximal (access) and distal (deep) binding pockets (abbreviated PBP and DBP, respectively), which are separated by the gate G-loop, also known as a switch loop (Eicher et al. 2012, Nakashima et al. 2011, 2013, Tam et al. 2021, Zwama et al. 2018). There are a number of substrate channels that lead to the PBP and DBP, with some of them originating from the membrane leaflet (channels 1 and 4), while others (channels 2 and 3) syphon the soluble substrates directly from the periplasmic space (Fig. 4; Oswald, et al. 2016, Tam et al. 2020, Zwama et al. 2018).

The availability of the numerous channels with varied specificities, alongside the distinct multidrug-binding pockets helps explain the remarkably wide substrate spectrum of the RND pumps, including MtrD (Bolla et al. 2014, Chitsaz et al. 2019, Eicher et al. 2012, Kobayashi et al. 2014, Lyu et al. 2020, Nakashima et al. 2011, Ramaswamy et al. 2017, Tam et al. 2020, 2021, Zwama et al. 2018). The majority of the substrates are thought to be first vetted in the PBP, and then transferred to the DBP, which is facilitated by the gating G-loop (Eicher et al. 2012, Fairweather et al. 2021, Vargiu and Nikaido 2012), although large molecular weight drugs may be able to bypass the DBP altogether (Tam et al. 2021). The RND pumps, including MtrD utilize a functionally rotating mechanism within the functionally assembled trimer, coupling three different conformational states, known as Access (A)/Loose (L), Bound (B)/Tight (T), and Extrusion (E)/Open (O) conformer respectively based on the capability of the individual protomers to bind the drug, keeping it associated with the protomer and being able to release it into the receiving OMF channel (Murakami et al. 2006, Seeger et al. 2006). These conformer motions are allosterically linked to substrate binding, PAP association and proton acceptance and release to ensure a directionality of cycling and effective substrate expulsion (Alav et al. 2021).

### The MFS superfamily

This superfamily has members in all domains of life, working as uniporters, symporters, or antiporters (Huang et al. 1999). These proteins are generally highly hydrophobic and are predicted to be formed by 12 α-helices and short loops (Foster et al. 1983). The minimum functional structural unit seems to be a monomer, but the existence of homo-oligomers has also been proposed (Yin et al. 2006). Some of them interact with other periplasmic and OM proteins, as in the case of the EmrAB–TolC system. Members of this superfamily are powered by the PMF, although alternative coupling ion energy has also been proposed (Krulwich et al. 2005). The MFS efflux pump EmrAB–TolC from *E. coli* and the RosAB pump from *Yersinia enterocolitica* are involved in conferring the AMP resistance phenotype (Table 2; Bengoechea and Skurnik 2000, Weatherspoon-Griffin et al. 2014).

### K+ transporters (SKT)

One last system related to AMP resistance is the TrkG/H–TrkA potassium uptake system, widely found in bacteria and archaea, with the uptake of K^+^ linked to H^+^ symport (Durell et al. 1999). The homodimer TrkH, belonging to the superfamily of K+ transporters (SKTs), is a transmembrane protein responsible for the transport of K^+^ across the cell membrane (Bakker 1993, Cao et al. 2011). They coassemble with TrkA, a cytosolic partner protein containing NAD binding sites (Cao et al. 2013). The structures of the TrkH–TrkA complex in the presence of ADP or ATP have been recently reported (Zhang et al. 2020). In *E. coli*, the activity of the system is dependent on the association with the ATP-binding protein TrkE (also known as SapD), which also forms a part of the SapABCDF ABC transporter (Sugiyama et al. 2016). A *trkA* mutant has been shown to confer resistance against polymyxin B and protamine in *Vibrio vulnificus* (Table 2; Chen et al. 2004).

In summary, two main mechanisms of efflux are used by Gram-negative bacteria to confer resistance to AMPs: (1) the efflux of AMPs to the extracellular space, as exemplified by MtrCDE or AcrAB–TolC and (2) the internalization of AMPs to the cytoplasm with subsequent proteolytic degradation, exemplified by the action of the Sap-transporter system (Fig. 3).

In the next sections, we will discuss these systems in more detail and delve into the evidence linking them to the AMP-resistant phenotype in Gram-negative bacteria.

## The MtrCDE efflux pump confers AMP resistance

MtrCDE is a tripartite efflux pump in *N. gonorrhoeae*, which confers broad-spectrum resistance to structurally diverse antimicrobial molecules such as AMPs, nonionic detergents, fatty acids, bile salts, macrolides, β-lactams, tetracycline, and the extended-spectrum cephalosporin ceftriaxone, which is the last option for gonorrhoea therapy (Golparian et al. 2014, Hagman et al. 1995, Shafer et al. 1998).

There is mounting evidence that the MtrCDE pump is capable of recognition and effluxing of AMPs, utilizing similar mechanisms to the ones described above. Indeed, in *N. gonorrhoeae*, the isogenic transformant strains bearing insertional inactivated *mtrC*, or *mtrE* genes, and a 10-bp deletion at the 3´-end of the *mtrD* gene resulting in a MtrD truncation (strain BR54) were significantly more susceptible to PG-1 than the parental strain FA19 (Shafer et al. 1998). In addition, an inactivated *mtrD* affecting *mtrE* expression as well (*mtrD/mtrE* deficient strain) was more susceptible to the structurally diverse peptides PG-1, PC-8, and LL-37.

In the same work, the importance of the electrostatic interactions as a first step in the AMP-membrane interaction mechanism was shown, since both the FA19 and *mtrD/mtrE* deficient strain were more sensitive to PG-1 and LL-37 under low salt conditions, showing the *mtrD/mtrE* deficient strain higher susceptibility (Shafer et al. 1998). At low salt concentration, the *mtrD/mtrE* deficient strain was also susceptible to PC-8, a linearized PG-1 synthetic variant lacking both disulfide bonds, and this AMP was almost inactive against the FA19 strain. This correlates with the hypothesis that the intramolecular disulfide bonds are crucial for PG-1 activity (Qu et al. 1997). Unexpectedly, the *mtrD*/*mtrE* inactivated strain showed resistance to the human HNP-2 and rabbit NP-2 α-defensins at any salt concentration (Table 2; Shafer et al. 1998). These results suggest that the MtrCDE system in general, and the MtrD transporter in particular, is not involved in the efflux of defensins, but confers resistance to LL-37, although both are found in the genitourinary mucosae where they are involved in host defence mechanisms (Feng et al. 2003). The MtrCDE role was confirmed by testing deficient efflux pump mutants in a female mouse model of genital tract infection (Jerse et al. 2003).

In Warner et al. (2008), the *N. gonorrhoeae mtrR* mutants were consistently more resistant to LL-37 and its murine homologue CRAMP-38 than the WT strain (Table 2). Also a *mtrE:cat* mutant designed by using a natural *mtrR* mutant as the parental strain (MS11) showed a higher susceptibility to these AMPs. The authors confirmed the MtrCDE role in AMP efflux since mutations in *mtrCDE* reduced gonococcal survival in the female murine genital tract, and mutations causing derepression of the *mtrCDE* operon enhanced gonococcal survival (Warner et al. 2008).

In a work using different antimicrobial proteins and peptides released by neutrophils, the MtrCDE specific defence mechanism was shown to be location- and component-specific depending on the molecule to be exported (Handing et al. 2018). LL-37 was proved to be MtrCDE-dependent, since the *mtrD* and *mtrE* mutants were significantly more sensitive to killing than the parental strain (Table 2). Conversely, the glycopeptide vancomycin showed an MtrD-dependent but MtrE-independent sensitivity since the *mtrC* and *mtrD* mutants had a significantly lower MIC compared with the parent or *mtrE* mutant strains (Handing et al. 2018).

A third phenotype was shown in these experiments when using bigger cationic proteins as molecules to be extruded by efflux pumps. The 55 kDa bactericidal permeability-increasing protein (Elsbach 1998) was shown to be MtrE-dependent but MtrD-independent, since the *mtrE*, but not the *mtrD* mutant, was more sensitive to killing. The authors concluded that the Mtr system contributes to gonococcal survival after neutrophil challenge.

Transport of the cyclic peptides also appears to be MtrD-dependent, as the deletion of *mtrD* from *N. gonorrhoeae* FA19 resulted in a 2-fold reduction of the MICs for polymyxin B and colistin. The reduction in MIC for colistin was 4-fold in a KH15 background, maybe due to the higher level of *mtrCDE* expression in this strain due to the upregulation of the system by a single-base-pair deletion in the *mtrR* promoter (Table 2; Chitsaz et al. 2019), indicating that colistin B is an actively effluxed substrate of the pump. The MIC values were restored in the KH15 *ΔmtrD ΔnorM* derivative by reinsertion of *mtrD*. Mutagenesis studies in two aromatic residues located in the deep drug-binding pocket of MtrD (F176A and F623A) and in its switch loop (F612A) highlighted the importance of these residues for the binding to polymyxin B and other substrates (Chitsaz et al. 2019). ​​The amino acids R714 and K823, engaged in the entrance and proximal substrate binding site within the periplasmic domain of MtrD, were also shown to be critical for polymyxin B resistance when MtrCDE is overexpressed in a KH15 background (Lyu et al. 2020).

By using a library of mariner transposon mutants generated in an *N. meningitidis* strain, mutations within *mtrD* or *lptA* genes and the *pilMNOPQ* operon showed increased susceptibility to the cyclic polymyxin B, the α-helical LL-37 and the β-sheet protegrin-1 (Table   2; Tzeng et al. 2005). Thus, the AMP resistance in *N. meningitidis* was shown to involve multiple mechanisms including the MtrCDE efflux pump, lipid A modification as well as the type IV pili secretion system and the major OM porin PorB (Tzeng et al. 2005). The heteroresistance to polymyxin B, colistin, and LL-37 has also been recently observed in clinical isolates of *N. meningitidis* urethritis (Tzeng et al. 2019).

In 2007, *H. ducreyi* was shown to be naturally more resistant than *E. coli* to killing by LL-37, the α-defensins (HPN-1, HNP-2, HNP-3, and HD-5) and β-defensins (HBD-2, -3, and -4), AMPs that *H. ducreyi* can encounter during infection, being still susceptible to be killed by the nonrelated PG-1 (Mount et al. 2007). Some years later, an orthologue of the MtrCDE efflux pump was identified in *H. ducreyi*, showing high similarity with the MtrD (31% identical and 37% similar) and MtrC (29% identical and 44% similar) proteins in *N. gonorrhoeae* (Rinker et al. 2011). In *H. ducreyi*, the deletion of the PAP *mtrC* rendered the bacteria more sensitive to LL-37 and β-defensins (especially against HBD-3), but not to the α-defensins (Table 2; Rinker et al. 2011). The *mtrC* deletion also affected the OM protein profile, colony morphology, and activated the two-component system CpxRA. Despite the action of CpxRA, the authors showed that MtrCDE contributed to LL-37 and HBD-3 resistance in a CpxRA-independent way, with the MtrCDE transporter being the major determinant of resistance to HBD-3 in *H. ducreyi* (Rinker et al. 2011).

In any case, the mechanism of LL-37 resistance in *H. ducreyi* was shown to be multifactorial, since MtrCDE, the Sap transporter, and the Cpx regulon contribute to the resistant phenotype (Mount et al. 2010, Rinker et al. 2011). In spite of the *H. ducreyi* MtrCDE apparent ability to efflux β- but not α-defensins, it was suggested that the MtrCDE pump could also efflux α-defensins if increasing the peptide concentration (Rinker et al. 2011). Also, the existence of another main mechanism conferring resistance against α-defensins and masking the MtrCDE activity could not be ruled out. Some years later, cell envelope modification through the addition of PEA to the lipid A and the core oligosaccharide of LPS, was shown to be the main mechanism responsible for the α-defensin resistance (HD-5), also affecting β-defensin resistance (HBD-3), but not resistance to LL-37 (Trombley et al. 2015).

## The AcrAB–TolC system

Similar to the aforementioned MtrCDE system, the AcrAB–TolC efflux pump has a wide range of substrates including dyes, detergents, different classes of antibiotics, and solvents (Figs 3A and 4; Anes et al. 2015, Kobylka et al. 2020). AcrAB–TolC has been shown to be involved in conferring AMP resistance in *K. pneumoniae* and *Y. pestis* although this is contentious in *E. coli*.

In *K. pneumoniae*, the *acrB* knockout mutant was significantly more susceptible to polymyxin B, α-defensin HNP-1, and β-defensins HBD-1 and HBD-2 (Table 2; Padilla et al. 2010). The authors confirmed that this susceptibility was not due to a reduced expression of the capsular polysaccharide or LPS, since the WT as well as the *acrB* knockout mutant expressed similar amounts of both polysaccharides (Padilla et al. 2010). Finally, the authors tested the ability of the *acrB* knockout to cause pneumonia in a mouse model, showing that lungs from mice infected with the *acrB* knockout presented significant lower bacterial loads than those infected with the WT strain (Padilla et al. 2010).

In *Y. pestis*, the *ΔacrAB* and *ΔtolC* mutants were more sensitive to polymyxin B than the WT, suggesting that polymyxin B is a substrate for AcrAB–TolC (Table 2). As the *tolC* deletion dramatically increased the susceptibility to polymyxin B, it was proposed that other pumps could also efflux polymyxin B by using TolC as the exit duct (Lister et al. 2012).

In *E. coli*, the involvement of AcrAB–TolC in the AMP resistance phenotype is controversial. In 2009, using *E. coli* strains with *acrAB* overexpressed or inactivated, it was shown that LL-37, polymyxin B, α-defensins HNP-1–3 and HD-5, and β-defensin hBD-2 were not substrates of AcrAB–TolC (Table 2; Rieg et al. 2009). However, in 2010, Warner and Levy suggested that LL-37, polymyxin B, and the β-defensin HBD-1 were substrates of AcrAB–TolC since the *acrAB* deficient mutant showed susceptibility to these AMPs (Table 2; Warner and Levy 2010). As this susceptibility was even greater in the *tolC* deficient mutant, they proposed the existence of other efflux pumps using TolC as a mediator of the AMP efflux [as seen later for *Y. pestis* (Lister et al. 2012)]. The susceptibility to the defensins was increased in a *tolC* mutant, while an *acrAB* deletion mutant only showed an increase in susceptibility to β-defensin (HBD-1) but not to the α-defensin HNP-2.

When using the *acrAB* and *tolC* mutants in the presence of the PMF inhibitor carbonyl cyanide-m-chlorophenylhydrazone (CCCP), the levels of LL-37 susceptibility increased in the a*crAB* mutant. Surprisingly, the strain containing the *tolC* deletion caused a 70-fold decrease in susceptibility compared to the non-CCCP-treated control. The authors proposed two hypotheses to explain this fact: (1) the presence of another AMP uptake system, which is normally masked by other TolC efflux pumps, or (2) the loss of PMF could alter the OM charge such that the AMPs were less attracted to the membrane (Warner and Levy 2010).

The differences obtained by Rieg et al. (2009) and Warner and Levy (2010) were shown to be related to the use of different microbiological media for the experiments because the different ion concentrations of the media could affect the AMP stability and ability to bind negatively charged surfaces (D'Amato et al. 1975, Dorschner et al. 2006). This hypothesis was confirmed by Warner and Levy (2010) when performing parallel experiments with both media (MH broth and LB broth) comparing the AMP susceptibility for the WT, *acrAB*, and *tolC* mutants.

Since the deletion of *tolC* resulted in an increase in the levels of the transcriptional regulators MarA, SoxS, and Rob, and porins are among the many genes regulated by these transcriptional factors, Warner et al. proposed that the loss of AcrAB–TolC induced changes in membrane permeability beyond simply loss of efflux, which may also affect susceptibility (Saw et al. 2016, Warner and Levy 2010).

In 2014, it was shown that the deletion of *acrB*, as well as *emrB* in *E. coli*, rendered the bacteria more susceptible to protamine when compared with the isogenic WT strain, with the *tolC* mutant showing a more extreme phenotype (Table 2; Weatherspoon-Griffin et al. 2014). This observation suggested that an additional TolC-dependent efflux pump(s) contributes to the protamine resistance. In this work, the Δ*acrD*, Δ*acrE*, Δ*acrF*, Δ*mdtE*, Δ*mdtF*, Δ*macA*, Δ*macB*, Δ*emrK*, and Δ*emrY* single mutants showed the same survival rate than the WT strain when exposed to protamine. Thus, AcrAB–TolC and EmrAB–TolC should contribute to TolC-dependent protamine resistance (Weatherspoon-Griffin et al. 2014). An interesting CpxR-dependent regulation was proposed for AMP resistance in *E. coli*, in which the two component system CpxR/CpxA activates (1) the transcription of the *mar* operon, which induces the expression of the tripartite multidrug efflux transporters, and (2) the transcription of the *aroK* gene, enhancing the production of metabolites able to release the repressor MarR from the *marO* site increasing *marA* expression and synthesis of efflux pumps (Weatherspoon-Griffin et al. 2014).

## Other RND-efflux pumps implicated in the AMP-resistance phenotype

In *C. jejuni* NCTC 11168, a single mutant affecting *cmeE*, the IM component of the CmeDEF efflux pump showed a 2-fold increase in the susceptibility to polymyxin B, but not to protamine. The same result was obtained for the 21190 strain isolated from chicken, and for the *cmeF/cmeB* double mutants, which also lack the IM component of the main efflux pump CmeABC (Akiba et al. 2006).

In VexAB–TolC, a RND-efflux pump in *V. cholerae*, the Δ*vexB* strain, as well as the Δ*tolC*, were shown to decrease the MIC for polymyxin B (Table 2; Bina et al. 2008).

Recently, a new locus affecting the resistance to colistin has been identified in *K. pneumoniae*. The locus, called H239_3064 was predicted to be an RND-type efflux pump by homology, exhibiting a 49% amino acid identity with AcrB in *K. pneumoniae*, with unknown periplasmic adaptor and OM proteins. Deletion of H239_3064 resulted in an 8-fold decrease in the MIC of colistin. However, it is not clear if this pump directly performs the efflux of colistin or the efflux of substrates that affect the bacterial surface charge (Table 2; Cheng et al. 2018).

In spite of the high similarity between MexA and AcrA, or MexB and AcrB (71%, and 89%, respectively), the *P. aeruginosa* MexAB–OprM pump seems to be only linked to the AMP resistance phenotype when biofilms are formed (Table 2). The authors suggested that colistin could have an intracellular target in addition to its membrane interfering activity (Pamp et al. 2008). However, it seems that Rieg's experiments (done in MH media) were not repeated using LB media, raising the possibility that the media could also selectively affect AMP resistance conferred by *P. aeruginosa* MexAB–OprM (Rieg et al. 2009, Warner and Levy 2010). Also other efflux pumps in *P. aeruginosa* such as MexCD–OprJ and MexXY–OprM were not able to extrude polymyxin B (Masuda et al.^2000^).

## Sap system: importing and degrading AMPs as mechanism of resistance

In addition to the strategy of pumping AMPs out of the bacterial cell, another approach that some bacteria use to become AMP resistant is to transport the AMPs into the cytoplasm, where they are degraded. In Gram-negative bacteria, this transport is carried out by the ABC family transporter encoded by the *sapABCDF* operon (sensitive-to-antimicrobial-peptides; Groisman et al. 1992, López-Solanilla et al. 1998, Mason et al. 2005, 2006, 2011, McCoy et al. 2001, Parra-Lopez et al. 1993, 1994, Shelton et al. 2011). The multimeric system is composed of SapA acting as a periplasmic solute-binding protein, SapB and SapC as transmembrane proteins forming a pore in the IM, SapD, and SapF as ATPase subunits and SapZ as an integral membrane protein presumably associated with SapC (Parra-Lopez et al. 1993). SapA directly binds the AMP and shuttles it from the periplasm to the SapBCDF for transport into the bacterial cytoplasm, where the AMPs are further degraded and its amino acids are recycled (Mount et al. 2010, Parra-Lopez et al. 1993). This mechanism was confirmed in the NTHI by using HBD-3 and LL-37, showing that kinetics of uptake and cytoplasmic proteolytic degradation seem to be dependent on AMP structure and charge (Table 2; Shelton et al. 2011). In addition, the accumulation of AMPs in the periplasm of *sapBC* permease-deficient cells supported the mechanism whose main goal seems to be to avoid the direct interaction between the AMPs and the cytoplasmic membrane, which could be lethal for the bacteria (Shelton et al. 2011).

Another proof of AMP-SapA binding is related to the ability of the sap system to uptake iron-containing nutrients (e.g. heme) required for NTHI growth and survival. The heme–SapA binding was shown to be displaced by HBD-1, HBD-2, HBD-3, LL-37, HNP-1, and melittin (Table 2; Mason et al. 2011). The ability to bind and displace the heme group was proportional to the relative charge of the AMP, with HBD-3 being the most efficient (+11 net charge). This AMP ability to displace the heme group in SapA shows a hierarchy, where immune evasion supersedes the need for the iron acquisition function by the Sap system (Mason et al. 2011).

**Figure 5.**
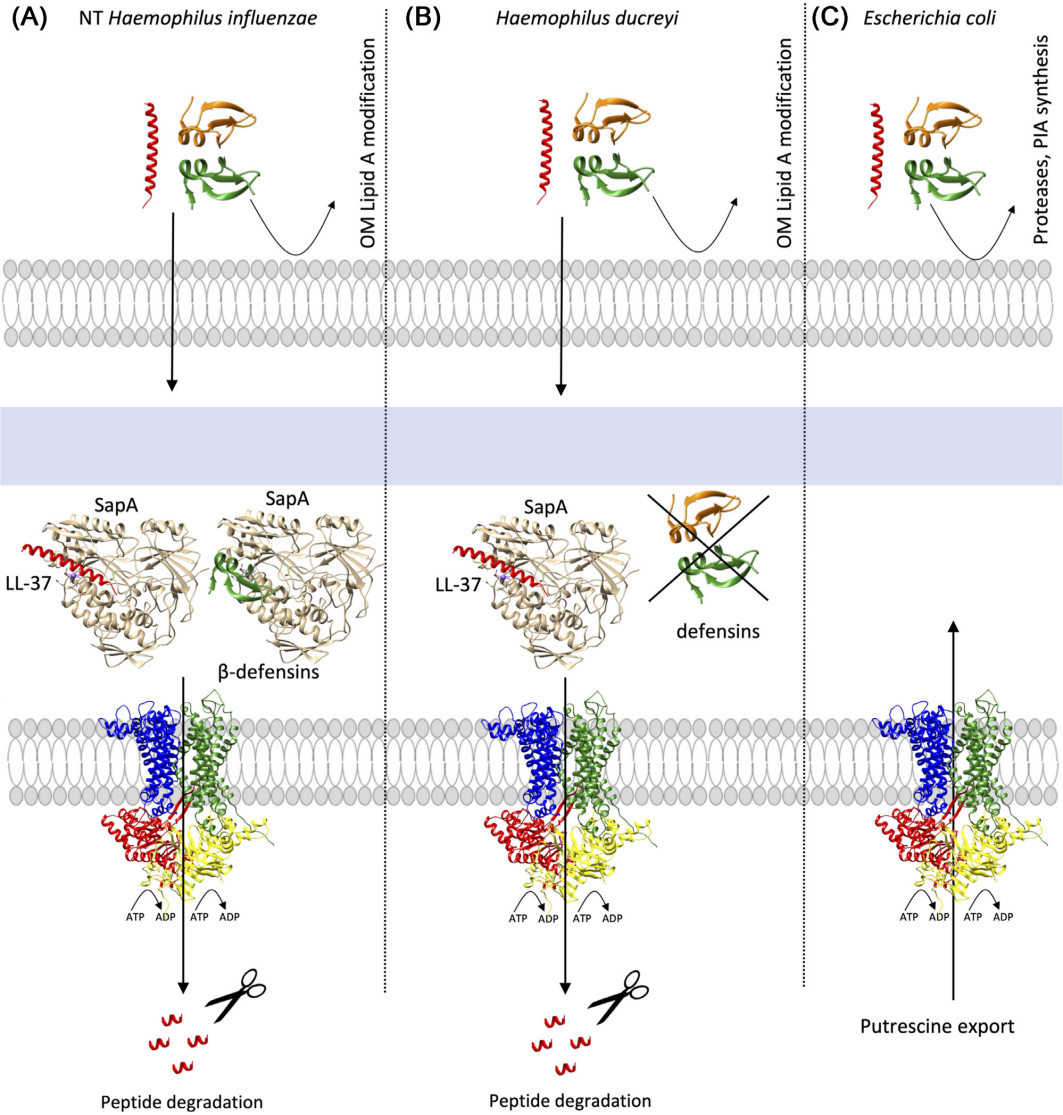
Representation of the Sap transporters in different microorganisms showing that behaviour against AMPs in the Sap transporters is microorganism-dependent. (A) The sap transporter in NTHI can introduce AMPs to the cytoplasm to be degraded. (B) The Sap transporter in *H. ducreyi* can introduce LL-37 into the cytoplasm but not defensins. In this microorganism, the PEA transferase genes confer resistance to the α-defensin HD-5 and the β-defensin HBD-3 but not to cathelicidins, such as LL-37. (C) The Sap transporter in *E. coli* is not related to AMP transport. LL-37 and defensins are represented as red helices and green/orange ribbons, respectively. The figure contains a model of the *E. coli* Sap transporter (this review) showing SapB in blue, SapC in green, SapD in red, and SapF in yellow (Fig. 3 for details).

Recently, the *H. influenzae* SapA protein structure has been solved in an open (no ligand) and closed conformation (Figs 3B and 5; Lukacik et al. 2021). These structures show a cavity volume that could accommodate a small ligand such as a short peptide or an extended polypeptide chain that could protrude out of the narrow openings of the SapA ligand-binding cavity, but with no space to accommodate AMPs in their folded state (Lukacik et al. 2021). In addition, the cavity is formed mainly by hydrophobic or neutral residues, showing a lack of countercharges to accommodate the AMPs. Moreover, the authors did not find any crystallographic or biophysical evidence of the highly purified SapA protein being able to bind hBD1, hBD2, hBD3, or LL-37 (Lukacik et al. 2021).

In *H. ducreyi*, the multimeric Sap transporter was shown to be responsible for LL-37 resistance but the *sapA* nonpolar mutant did not confer any resistance towards α- and β-defensins (Fig. 5B; Mount et al. 2010). A nonpolar *sapBC* mutant lacking both IM permeases of the Sap transporter, exhibited greater sensitivity than the *sapA* mutant to killing by LL-37, but it did not affect the resistance of *H. ducreyi* to human defensins (Table 2; Rinker et al. 2012). The lack of sensitivity against defensins exhibited by the Sap transporter was also confirmed by the inactivation of the *sapA* locus in *E. chrysanthemi* (renamed to *Dickeya dadantii* in 2005), an important phytopathogenic bacterium (Table 2; López-Solanilla et al. 1998). In addition, the SapBC channel in *H. ducreyi* retained activity when *sapA* is removed, suggesting that the specificity of the Sap system does not rely exclusively on the interaction with the periplasmic solute-binding SapA, and raising the idea that other unknown SapA-independent mechanisms can exist in *H. ducreyi*(Rinker et al. 2012). This mechanism could be the interaction with other periplasmic solute-binding components different to SapA or even the efflux of AMPs by MtrCDE (Letoffe et al. 2006).

As seen, the *H. ducreyi* Sap transporter cannot confer the resistant phenotype when attacked by defensins. In contrast, the *sapA* nonpolar mutant in the NTHI Sap transporter was approximately 8-fold more sensitive than the parent strain to killing by recombinant chinchilla β-defensin-1 (cBD-1), an orthologue of human β-defensin-3 (HBD-3; Fig. 5A and Table 2; Mason et al. 2005). In addition, it showed a significantly attenuated ability to survive in a chinchilla model of otitis media compared with the parent strain (Mason et al. 2005). Also the *H. influenzae sapD* mutant was sensitive to killing by cDB-1, HBD-3, and LL-37 (Mason et al. 2006). In addition, HBD-2, HBD-3, LL-37, hNP-1, and melittin were shown to be able to bind and displace the heme group bound to SapA. They were also shown to be susceptible to degradation by cytoplasmic proteolysis (Table 2; Mason et al. 2011, Shelton et al. 2011).

In *H. ducreyi*, the modification of Lipid A in the OM confers resistance by means of electrostatic repulsion. Specifically, the phosphoethanolamine (PEA) transferase genes confer resistance to the α-defensin HD-5 and the β-defensin HBD-3, but not to cathelicidins, such as LL-37 (Trombley et al. 2015).The resistance to LL-37 would come from the MtrCDE efflux pump and Sap transporter activity, and these two mechanisms could mask others such as the PEA modification (Trombley et al. 2015). In the case of NTHI, low concentrations of AMPs (including LL-37) could be counteracted by the modification of the OM (Lysenko et al. 2000), but increasing concentrations would increase the production of the Sap transporter, with the corresponding binding of SapA to the AMP and cytoplasmic membrane transport for the proteolytic degradation in the cytoplasm (Shelton et al. 2011).

Although the main mechanism of resistance to AMP attack in *P. mirabilis* is LPS modification, the *sapD* mutant showed that the Sap transporter is also involved in conferring resistance to polymyxin B, but not to the β-sheet protegrin, its analogue IB-367, nisin, tachytegrin A, and polyphemusin (Table 2; McCoy et al. 2001). In *A. pleuropneumoniae*, a Gram-negative bacterial pathogen responsible for porcine pleuropneumonia, the *ΔsapA* mutant showed increased sensitivity to PR-39, a linear porcine AMP with a high proline content (Table 2; Xie et al. 2017). In *Vibrio fischeri*, a marine Gram-negative bacteria, the polar mutation within the *sapABCDF* operon does not confer resistance to LL-37, polymyxin, protamine, the indolicidin derivative CP11CN, and the hybrid cecropin/melittin CP26, CP28, and CP29 peptides, but seems to be required for normal growth (Table 2; Chen et al. 2000, Lupp et al. 2002).

Groisman et al. (1992) identified eight distinct protamine resistance loci in a collection of *S*. Typhimurium mutants. The *sapC* and *sapD* mutants were also shown to confer resistance to melittin, and the crude granulocyte extract (Table 2; Groisman et al. 1992, Parra-Lopez et al. 1993). Also, the *sapG* gene was shown to be a NAD+ binding protein 99% identical to the *E. coli* low-affinity potassium uptake component TrkA, showing the *sapG* mutants a hypersensitivity to protamine (Table 2; Groisman et al. 1992, Parra-Lopez et al. 1994). In this paper, the authors proposed that SapG, SapJ, and the SapABCDF transporter function as a complex to mediate both peptide and K^+^ transport (Parra-Lopez et al. 1994). In this complex, SapABCDF would be the peptide pore while SapJ (also known as TrkH) would form the K^+^ pore, although an alternative model in which both SapJ and SapABCDF form a single pore for K+ and peptide transport could not be ruled out. In this model, SapG would act to coordinate the peptide and K+ transport functions of the complex (Parra-Lopez et al. 1994), as shown by the *E. coli* TrkH protein which is dependent on the ATP-binding protein SapD (also known as TrkE), which is part of the SapABCDF ABC transporter, although not all the Trk systems are dependent on SapD and can utilize AP-binding proteins from other ABC transporters (Nakamura et al. 1998).

In *Vibrio*, the potassium uptake system consists of two proteins: the integral membrane K^+^-translocating protein TrkH (or TrkG), and the NAD-binding peripheral membrane protein TrkA (Bakker 1993, Cao et al. 2011). The *trkA* inactivated mutant showed a higher sensitivity to protamine and polymyxin B compared with the WT strain (Table 2; Chen et al. 2004).

An important exception occurs in *E. coli*, whose Sap transporter does not seem to be involved in conferring the AMP resistant phenotype since the *sapBCDF* knockout strain did not confer resistance to LL-37 (Fig. 5C and Table 2; Sugiyama et al. 2016). This fact is striking since it was reported that the Δ*sapABCDF* strain in *S*. Typhimurium was more sensitive to protamine than the parental strain (Parra-Lopez et al. 1993). While the amino acid identity of SapABCDF in *Salmonella* and *E. coli* is very high (between 90% and 98% for all the genes in the operon), its gene organization is different since *sapABCDF* in *Salmonella* are expressed polycistronically, but in *E. coli* the promoter of *sapA* is located independently of that of *sapBCDF* (Sugiyama et al. 2016). In addition, the predicted sigma factor is different for *sapA* and *sapBCDF* (σ70 and σ54, respectively; Sugiyama et al. 2016). In this work, the authors succeed in assigning a function related to putrescine export to the *E. coli* Sap transporter (Sugiyama et al. 2016). In *E. coli*, other resistant mechanisms, such as the synthesis of PIA or the activity of the omptin family of aspartate proteases seem to be responsible for AMP resistance (Stumpe et al. 1998, Thomassin et al. 2012, Wang et al. 2004).

## Other ABC-transporters implicated in the AMP-resistance phenotype

The ABC transporter YejABEF is composed of four genes: *yejA*, which encodes a putative periplasmic binding protein; *yejB* and *yejE*, which encode putative permease components; and *yejF*, which encodes the ATPase component of this transporter (Eswarappa et al. 2008). Partial and complete deletion of the operon in *Brucella melitensis* showed a significantly increased sensitivity to acidic stress. Δ*yejE* and Δ*yejABEF* mutants were also more sensitive than the WT to polymyxin B (Table 2; Zhen Wang et al. 2016). Moreover, cell and mouse infection assays indicated that both deletions have restricted invasion and replication abilities inside macrophages. In *Salmonella*, the *yejF* knockout showed increased sensitivity to protamine, melittin, polymyxin B, HBD-1, and HBD-2, and was compromised in its capacity to proliferate inside activated macrophages and epithelial cells (Table 2; Eswarappa et al. 2008).

In the tripartite ABC transporter MacAB–TolC, MacB hydrolyses cytoplasmic ATP and the molecules are translocated through MacA and TolC from the periplasm to the extracellular space (Crow et al. 2017, Fitzpatrick et al. 2017, Greene et al. 2018). Recently, a role in AMP resistance has been proposed for MacAB–TolC in *Salmonella* as constitutive expression of *macAB* conferred resistance against C18G, a synthetic α-helical peptide derived from human platelet factor IV (Table 2; Honeycutt et al.^2020^).

## MFS-efflux pumps implicated in the AMP-resistance phenotype

In *Y. enterocolitica, rosAB* encodes a temperature-regulated MFS efflux pump, i.e. coupled to a potassium antiporter (Table 2; Bengoechea and Skurnik 2000). This efflux pump has been shown to confer resistance against polymyxin B, cecropin P1 and melittin (Bengoechea and Skurnik 2000). The mechanism seems to involve the efflux of AMPs by the IM protein RosA after the AMPs enter the cytoplasm, using the energy provided by RosB. Once the AMPs reach the periplasmic space, an OM protein such as TolC would be needed to transport the substrates to the extracellular environment (Bengoechea and Skurnik 2000).

The tripartite *E. coli* EmrAB–TolC efflux pump, belonging to the MFS superfamily was shown to confer protamine resistance. The percentage of survival in the presence of protamine for the Δ*emrB* and Δ*tolC* mutants was 20% and 0%, respectively, suggesting that those deletions rendered bacteria more susceptible to protamine (Table 2; Weatherspoon-Griffin et al. 2014). Also in *Acinetobacter baumannii* the *emrB* knockout mutant was shown to be more susceptible to colistin than the WT strain by using time kill assays and minimal inhibitory concentration determination (Lin et al. 2017).

## Open questions

As shown in this review, Gram-negative bacteria have developed two main mechanisms to get rid of the AMPs using transport, including the efflux of AMPs to the extracellular space by tripartite efflux pumps including MtrCDE or AcrAB–TolC, and the internalization of AMPs to the cytoplasm and posterior proteolytic digestion, by the SapABCDF transporter.

In this section, we will expose some questions that arose while writing this review.

### How do efflux pumps recognize and process a broad variety of substrate peptides unrelated in structure and sequence?

The most intriguing question that comes from this review is how the efflux pumps can recognize a broad structural substrate range (e.g. the *N. gonorrhoeae* MtrCDE can efflux AMPs as different as the α-helical LL-37, the β-sheet PG-1, and the cyclic PXB). A potential explanation would be that IM proteins, such as MtrD or the *Staphylococcus aureus* phenol-soluble modulin (Pmt) transporter are supposed to be able to export peptides when fully inserted in the IM (Chang 2003, Cheung et al. 2018). In their membrane-bound conformation, the structural differences between the α-helical or β-sheet AMPs would be reduced since they would be restricted by the environmental constraints imposed by the phospholipid membrane. In a recent molecular dynamic simulation using a hBD-3 analog, it was revealed that the membrane-binding rigidifies the peptide, enhancing its structural polymorphism, and promoting β-strand conformation (Kang et al. 2019). Also other factors such as the presence of secondary metabolites or reactive oxygen species (ROS) could contribute to any structural similarities between AMPs. Besides being able to capture IM-inserted peptides, most tripartite efflux pumps are likely to intercept substrates from the periplasm or outer leaflet of the IM (Alav et al. 2021). In this case, an option to transport peptides differing in their 3D structures would be to transport them as unfolded peptides. Although there is no available information regarding unfolding mechanisms for AMPs, there is evidence of similar peptides (e.g. amyloid peptides Aβ-42 and Aβ-40) being transported in a partial or complete disordered state by the ABC transporter P-glycoprotein (McCormick et al. 2021). Regretfully, the authors in this study could not conclude with certainty whether P-glycoprotein was able to transport or disrupt folded Aβ monomers or whether it could facilitate the folding of the peptides during the transport process. Also the existence of the translocon complex component SecDF, a member of the RND superfamily, could support this hypothesis. In *E. coli*, SecDF moves proteins (including unfolded polypeptides and other diverse substrates) through the IM towards the periplasmic space (Rahman et al. 2017, Tsukazaki et al. 2011). Sec recognizes their substrates by a Sec signal that does not show sequence similarities, but contains a conserved tripartite overall structure consisting of a cationic N-terminal region, a central hydrophobic core, and a polar C-terminus (Rusch and Kendall 2007). By analysing the AMPs sequences, we could not find any conserved sequence, and even less a hydrophobic core, since AMPs are, by nature, amphipathic, but an unknown recognition sequence to direct the AMPs to the efflux pump cannot be ruled out. Finding this sequence would allow modification of AMPs to avoid their recognition by the efflux pump and posterior removal to the extracellular space.

Recently, the *S. aureus* ABC transporter PmtABCD, responsible for the secretion of all the known phenol soluble modulins (PSMs), has been shown to be also able to export important human AMPs such as LL-37 and hBD3 (R. Chatterjee et al. 2013, Cheung et al. 2018, Wang et al. 2007, Zeytuni et al. 2020). This dual transport is not difficult to understand since PSMs and AMPs share structural features [e.g. PSMα and LL-37 are helical and a high sequence similarity exists between the short PSMα3 and the core of LL-37 (Cheung et al. 2018, Engelberg and Landau 2020)]. As other substrates of the Pmt transporter are PSMβ3 and hBD3 with more complicated structures in solution, it is assumed that the transporter is also able to capture the membrane-inserted peptides as well (Cheung et al. 2018). This ability could be explained by the proposed ‘vacuum cleaner’ mechanism in which the efflux pumps can take hydrophobic molecules embedded in the membrane to efflux them (Chang 2003, Raviv et al. 1990). In this way, the Pmt transporter could accept membrane embedded peptides coming from the periplasm (AMPs) and PSMs coming from the cytoplasm (Cheung et al. 2018). A similar mechanism to accept AMPs coming from the cytoplasm cannot be ruled out for other efflux pumps.

As a general rule, it seems that any efflux pump is able to efflux almost all the structurally different AMPs, but that the effect is masked by other dominant mechanisms to avoid AMP attack (e.g. α-defensins are not effluxed by MtrCDE in *H. ducreyi* even though the pump is able to do it, because PEA modification is the main mechanism for α-defensin resistance; Trombley et al. 2015).

In the case of the AMP-SapA binding, it is assumed that the AMPs will be intercepted in the periplasm in their membrane-unbound conformation. Thus, the different AMP structures that SapA can bind (e.g. the NTHI SapA protein can bind the β-sheet HBD-3, the α-helix LL-37, and even the heme-group) could depend on the SapA general architecture (Shelton et al. 2011). The recent *H. influenzae* SapA protein structure solved in an open (no ligand) and closed conformation, shows a cavity volume (and lack of negative charge) unable to accommodate a complete folded AMP (Lukacik et al. 2021). The binding of the folded AMP protruding out of the narrow openings of the SapA ligand-binding cavity was proposed by the authors. Moreover, the authors could not obtain any SapA–AMP complexes when using pure SapA protein, but by contrast were able to obtain complexes of SapA bound to the heme group and dsRNA (Lukacik et al. 2021). This may suggest that an additional protein, acting as a chaperone, would be needed for the AMPs–SapA binding before transport to the cytoplasm by the SacBCDF transporter. This process may possibly be associated with at least partial unfolding of the AMPs to facilitate transport. Such a possibility is also supported by the fact that the *H. ducreyi* SapBC channel retains activity even when SapA is not present, suggesting that other unknown SapA-independent mechanisms may exist in this microorganism, including the AMP interaction with other periplasmic solute-binding components different to SapA (Rinker et al. 2012).

### How do efflux pumps process such large molecules as AMPs?

Another striking question is related to the size of the molecules to be extruded. Aside from the AMPs, the maximum molecule size transported by AcrAB–TolC are the macrolides, with a molecular weight smaller than 1000 Da (Ababou and Koronakis 2016, Tam et al. 2021). The cyclic AMPs colistin and polymyxin B are a similar size (approx MW 1200 Da), but this is not the case for bigger AMPs such as LL-37 (4493 Da) or defensins (3000-5000Da). So, how could they interact and pass through the narrow channels in the IM components (e.g. MtrD) of the efflux pumps?

Recently, a new path for high molecular weight drugs (e.g. macrolides and ansamycins) has been proposed in AcrB (Tam et al. 2021). In this path, the high molecular weight drugs would be initially captured in the access pocket (via channel 2), where the switch loop would accommodate their binding. After that, the drug would be accommodated in the deep binding pocket region, and subsequently be exported through the O protomer exit tunnel (Tam et al. 2021). As polymyxins share structural features with macrolides, it is tempting to think about a similar recognition mechanism.

Molecular dynamics studies have shown the movement of molecules inside efflux pumps (e.g. progesterone in the *N. gonorrhoeae* MtrCDE efflux pump; Chitsaz et al. 2019). The computational approach would be an interesting tool to clear up structural questions related to the AMP–efflux pumps complexes, with the limitations of using short AMPs in these simulations (Ramesh et al. 2016). Also, structures of the AMP–efflux pump IM protein could be obtained by structural approaches, such as X-ray crystallography, as done recently by crystallizing MexB in the presence of high-molecular-mass compounds (Sakurai et al. 2019). The structure of complexes containing AMPs have been obtained as in the case of proline-rich peptides bound to the *Thermus thermophilus* 70S ribosome or short AMPs (11–13 amino acids) bound to *P. aeruginosa* lectin Lec B (Baeriswyl et al. 2019, Gagnon et al. 2016).

### Are the OMF proteins potential entry channels for AMPs?

Loss of *mtrE* enhanced, not reduced, gonococcal survival after exposure to azurocidin (37 kDa) raising the question if MtrE could be a portal across the OM for some antimicrobials, although other possibilities related to secondary effects linked to *mtrE* loss could not be ruled out (e.g. the *tolC* deletion increases the activity of the transcriptional regulators MarA, SoxS, and Rob, being these regulators responsible for the porin regulation and producing potential changes in membrane permeability; Handing et al. 2018). The possibility that MtrE can act as an AMP entry portal is supported by the recent finding that TolC is able to import bacteriocins (MW 60 kDa) in Gram-negative bacteria (Housden et al. 2021). If the OM proteins act as AMP access channels, then AMP entry can possibly be potentiated by blocking the OM channel in open state as done by using MtrE mutants and vancomycin (MW 1449 Da; Janganan et al. 2013). Such approach was also employed for TolC, where the introduction of mutations caused an increased susceptibility to vancomycin suggesting that these mutations cause disruption of the OM permeability barrier at the level of TolC gating (Marshall and Bavro 2020). It is plausible that other cyclic AMPs such as polymyxin B or colistin could also use the OM as entry channel.

We could also take advantage of the ability of the SapABCDF transporter to introduce AMPs in the cytoplasm by introducing proteolytic resistant AMPs (Lu et al. 2020). Many efforts have been made to deal with this weakness of AMPs (e.g. D-amino acid substitutions, introduction of disulfide bonds, immobilize them on surfaces, use non natural amino acids incorporation, cyclization, and nano delivery systems), but further research is needed (Biswaro et al. 2018, Gentilucci et al. 2010, Jia et al. 2017).

### Could we use AMPs as scaffolds to design efflux pump inhibitors?

A significant global effort is underway to develop efflux pump inhibitors (EPIs) to potentiate the use of the existing antibiotics (Marshall et al. 2020). If we can better understand AMP–efflux pump interactions, similar strategies could be followed to design peptide-based EPIs. Thus, understanding the specific AMP–efflux pump interactions is critical for informed design of potential EPIs. While there are no current experimental 3D structures of efflux pumps in complex with AMPs, such approaches have been followed successfully in similar scenarios, e.g. by using pore-blocking toxins that inhibit voltage-dependent K+ channels (Banerjee et al. 2013). The 1-(1-naphthylmethyl)-piperazine (NMP), an AcrAB–TolC inhibitor, was shown to interact with the critically important residue (Phe610) within the deep binding pocket of AcrB and causing conformational change in AcrB (Bohnert and Kern 2005, Vargiu et al. 2014). The structure of the complex formed by AcrB and the pyridopyrimidine derivative inhibitor D13-9001 in *E. coli* and MexB of *P. aeruginosa* showed the binding of the inhibitor to the distal pocket, preventing the binding of the substrates (Nakashima et al. 2013), while the binding of phenylalanylarginine-β-naphthylamide (PAβN) and other inhibitors to AcrB has been shown by computational approaches (Vargiu et al. 2014). Importantly, pyranopyridines, such as MBX2319, which bind within a phenylalanine-rich cage that branches from the deep binding pocket of AcrB, form extensive hydrophobic interactions within it, which allowed for an effective computational derivation of the structure of the lead compounds, increasing further the affinity of interaction and providing a template for an effective pump inhibition (Sjuts et al. 2016).

In addition, the use of peptides able to change their conformation depending on the pH or temperature could be useful in order to plug a channel. For doing this, the use of self-assembling peptides, whose huge variety of structures depends on the environmental conditions, would be a good option (Lee et al. 2019, Lombardi et al. 2019). Furthermore, peptide tectons, defined as peptide building blocks exhibiting structural complementarity at the interacting interfaces, can self‐assemble into defined supramolecular structures promoted by these complementary interactions (Lou et al. 2019). Some of these supramolecular structures (e.g. peptide-based cages) have shown their ability in drug delivery while others (e.g. flexible fibres of indefinite length or large colloidal-scale assemblies) have been considered as new biomaterials with applications in biotechnology (Boyle et al. 2012, Fletcher et al. 2013).

Another approach would be to target and disrupt the dimerization interface as done by some peptide-based EPIs of the *P. aeruginosa* small multidrug resistance (SMR) efflux protein (Mitchell et al. 2019).

## Conclusions/future perspectives

Understanding in more detail the physical interaction between AMPs and efflux pumps/transporters will help us to develop novel strategies to take advantage of or inhibit the efflux process. First, the structural information of the complexes could guide us to design AMPs able to avoid the action of efflux pumps and/or proteolytic degradation in the cytoplasm. Second, the structural information could inspire the design of AMP-based EPIs able to plug the efflux pump channels. Third, we could design drugs fused to AMPs and use these peptides to transport them to the cytoplasm via the Sap transporter system. Once there, the drug could be released from the AMPs by bacterial proteases. Crystal structures of the AMP–efflux pump complexes will be crucial for the rational design of new drugs using these innovative approaches.

Lastly, by understanding the transport of the ribosomally synthesized and the nonribosomally synthesized AMPs we could answer two interesting questions. Future work on the nonribosomally synthesized AMPs, currently licenced for therapeutic use (e.g. colistin), should clarify whether efflux is a relevant mechanism of resistance to AMP-based medicines. On the other hand, future research into the ribosomally synthesized AMPs is required to understand how bacteria deal with the host immune response during infection. Given the emerging prominent role that efflux appears to play in resistance to both types of AMPs, efflux inhibitors have the potential to be an important addition to the physician's arsenal in the post antibiotic era.
